# Effects of high-volume online mixed-hemodiafiltration on anemia management in dialysis patients

**DOI:** 10.1371/journal.pone.0212795

**Published:** 2019-02-22

**Authors:** Luciano A. Pedrini, Adam M. Zawada, Anke C. Winter, Jenny Pham, Gudrun Klein, Melanie Wolf, Astrid Feuersenger, Pio Ruggiero, Annalisa Feliciani, Carlo Barbieri, Adelheid Gauly, Bernard Canaud, Stefano Stuard

**Affiliations:** 1 Department of Nephrology and Dialysis, NephroCare-ASST Bergamo-Est, Bolognini Hospital, Seriate, Italy; 2 Fresenius Medical Care Deutschland GmbH, Clinical and Epidemiological Research, Bad Homburg, Germany; 3 Fresenius Medical Care, Palazzo Pignano, Italy; 4 Fresenius Medical Care Deutschland GmbH, Medical Office EMEA, Bad Homburg, Germany; 5 Fresenius Medical Care Deutschland GmbH, Clinical &Therapeutical Governance, Bad Homburg, Germany; University of Mississippi Medical Center, UNITED STATES

## Abstract

**Background:**

Anemia is a major comorbidity of patients with end-stage renal disease and poses an enormous economic burden to health-care systems. High dose erythropoiesis-stimulating agents (ESAs) have been associated with unfavorable clinical outcomes. We explored whether mixed-dilution hemodiafiltration (Mixed-HDF), based on its innovative substitution modality, may improve anemia outcomes compared to the traditional post-dilution hemodiafiltration (Post-HDF).

**Methods:**

We included 174 adult prevalent dialysis patients (87 on Mixed-HDF, 87 on Post-HDF) treated in 24 NephroCare dialysis centers between January 2010 and August 2016 into this retrospective cohort study. All patients were dialyzed three times per week and had fistula/graft as vascular access. Patients were matched at baseline and followed over a one-year period. The courses of hemoglobin levels (Hb) and monthly ESA consumption were compared between the two groups with linear mixed models.

**Results:**

Mean baseline Hb was 11.9±1.3 and 11.8±1.1g/dl in patients on Mixed- and Post-HDF, respectively. While Hb remained stable in patients on Mixed-HDF, it decreased slightly in patients on Post-HDF (at month 12: 11.8±1.2 vs 11.1±1.2g/dl). This tendency was confirmed by our linear mixed model (p = 0.0514 for *treatment x time* interaction). Baseline median ESA consumption was 6000 [Q1:0;Q3:16000] IU/4 weeks in both groups. Throughout the observation period ESA doses tended to be lower in the Mixed-HDF group (4000 [Q1:0;Q3:16000] vs 8000 [Q1:0;Q3:20000] IU/4 weeks at month 12; p = 0.0791 for *treatment x time* interaction). Sensitivity analyses, adjusting for differences not covered by matching at baseline, strengthened our results (Hb: p = 0.0124; ESA: p = 0.0687).

**Conclusions:**

Results of our explorative study suggest that patients on Mixed-HDF may have clinical benefits in terms of anemia management. This may also have a beneficial economic impact. Future studies are needed to confirm our hypothesis-generating results and to provide additional evidence on the potential beneficial effects of Mixed-HDF.

## Introduction

In the past few years large controlled trials and meta-analyses suggested that on-line hemodiafiltration performed with high convective volume (HV-OL-HDF) may have substantial benefits in terms of survival of patients on chronic renal replacement therapy (RRT) [1–3]. The mechanisms by which this technique promotes such benefits are still debated. Several studies demonstrated that HDF improves uremic toxicity more efficiently than conventional HD by enhancing convective removal of protein-bound and middle molecular uremic toxins [4–7]. Pro-inflammatory cytokines and complement products are also more effectively removed in HDF with the effect of reducing chronic inflammation of dialysis patients [8–10]. Moreover, some evidence supports the hypothesis that patients on HDF may have benefits in terms of anemia management: lower erythropoiesis-stimulating agents (ESAs) dose was required in patients on HDF compared to those on conventional HD in order to maintain hemoglobin (Hb) levels within the recommended range [6, 11–13]. As well, lower ESA resistance index (ERI) was reported in patients treated with convective dialysis technique as compared to patients treated with conventional HD [14, 15]. However, data in the context of anemia management and treatment modality is conflicting as other studies did not find improved anemia parameters in patients treated with convective dialysis technique [1, 16–18].

HV-OL-HDF may be performed with different substitution modalities [19]. Currently, post-dilution HDF (Post-HDF) is considered the most efficient modality. However, this substitution mode may be challenging in certain patient groups, especially in patients with high predialysis hematocrit levels or low vascular access blood flow. Very high hematocrit may be achieved within the dialyzer capillaries in the attempt to force ultrafiltration and to obtain high convective volumes [20, 21]. The resulting hemoconcentration and hyper-viscosity coupled with increased shear stress and high pressure regimen inside the dialyzer is a known risk factor for red cell damage and hemolysis [21]. In Mixed-HDF, the pre- and post-dilution substitution rates are adjusted by means of a feedback control system to obtain the maximal filtration fraction within safe pressure and hydraulic conditions, thus preventing progressive hemoconcentration [22, 23].

Based on these properties, we hypothesized that Mixed-HDF might have a positive impact on anemia control. ESA treatment does not only pose a huge economic burden to health care systems, high ESA doses have been recently associated with severe negative outcomes, such as increased risk for cardiovascular complications, cancer progression and mortality [24–29]. Therefore, optimized dialysis modalities which may reduce ESA consumption may have a place among new strategies for anemia correction. In the present study we explored the impact of Mixed-HDF on hemoglobin levels and ESA consumption in a longitudinal approach, comparing the course of these parameters between patients treated with Mixed-HDF and patients treated with the traditional Post-HDF modality.

## Methods

### Study population

In this multicenter, retrospective cohort study, dialysis patients from 24 FME NephroCare dialysis centers in Italy (n = 21), Slovenia (n = 2) and Czech Republic (n = 1) were included. All centers share common treatment targets (e.g. Hb 10–12 g/dl). Dialysis centers were eligible for inclusion if at least 10 patients were treated with Mixed-HDF over ≥1 year.

Pseudonymized patient data were accessed through the European Clinical Database (EuCliD), which has been described elsewhere in detail [30]. EuCliD gathers medical information regarding demographics, medical history, underlying kidney disease and comorbidities, vascular access, dialysis treatment and medication as well as hospitalization and mortality data. All patients in the present study provided written informed consent for the use of their pseudonymized data for scientific research purposes.

**Fig 1:**
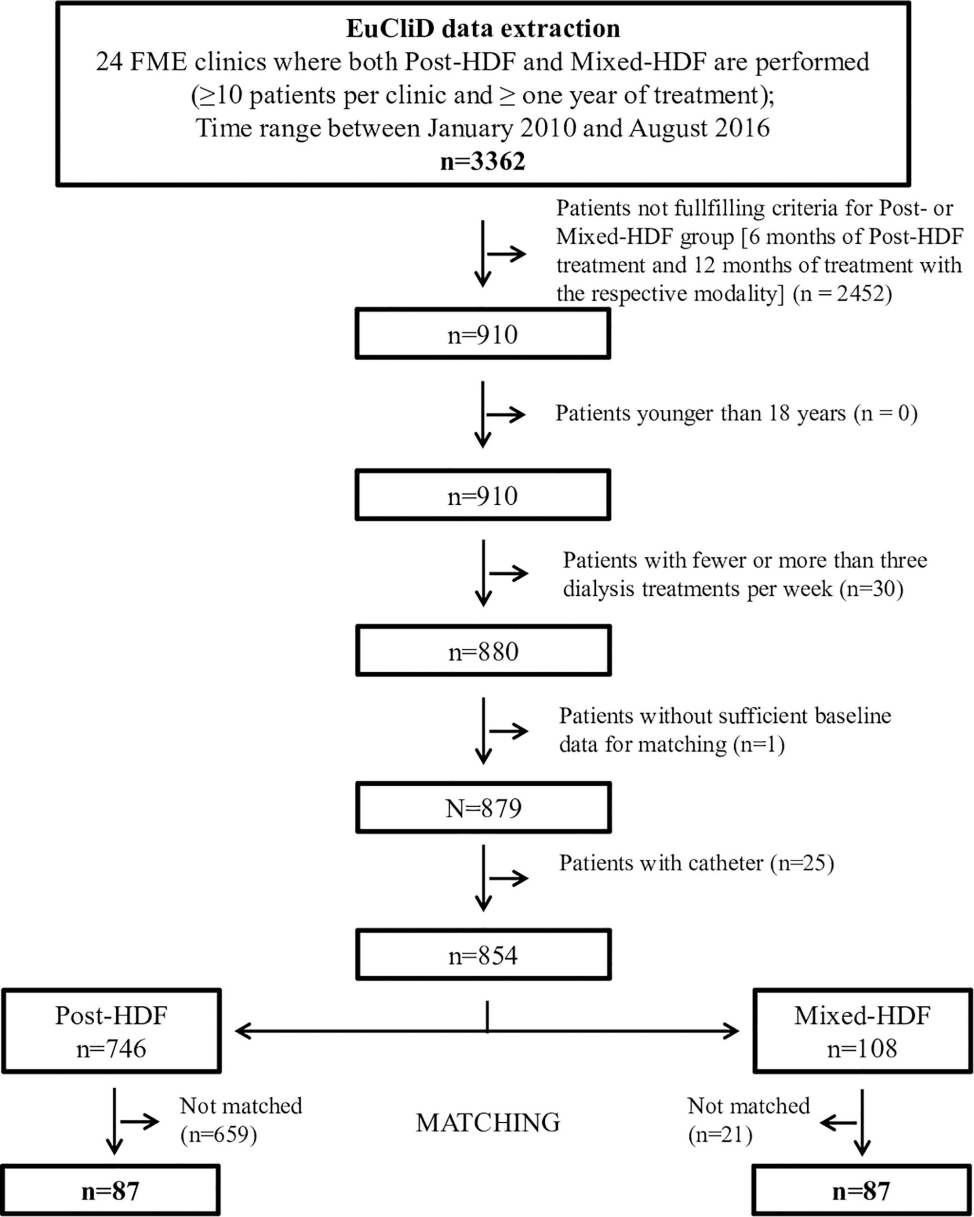
Flow chart with patient numbers. EuCliD: European Clinical Database, FME: Fresenius Medical Care, Mixed-HDF: Mixed-dilution hemodiafiltration, Post-HDF: Post-dilution hemodiafiltration.

Out of 3362 hemodialysis patients treated in the 24 NephroCare centers between January 2010 and August 2016, 854 adult patients (108 Mixed-HDF, 746 Post-HDF) with fistula/graft as vascular access and receiving three dialysis treatments per week were included into the patient pool used for matching (Fig 1).

### Exposure definition

**Fig 2:**
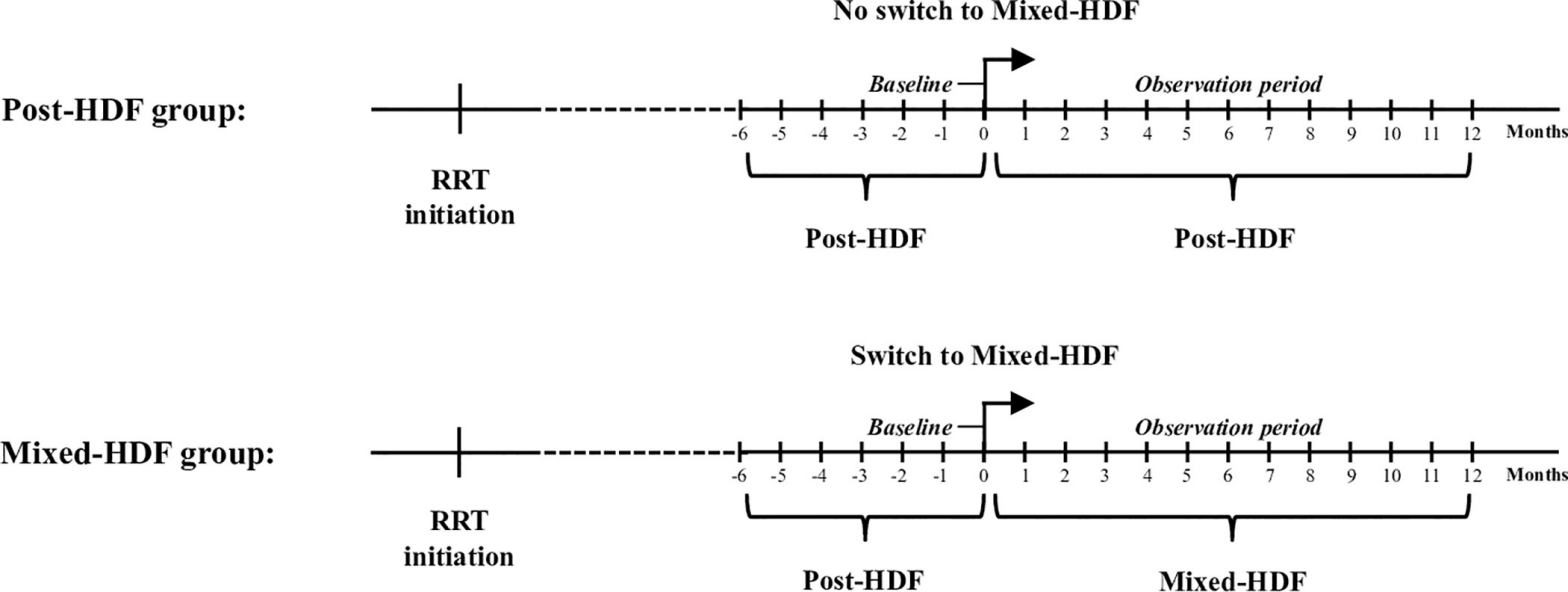
Study design. Mixed-HDF: Mixed-dilution hemodiafiltration, Post-HDF: Post-dilution hemodiafiltration, RRT: Renal replacement therapy.

Patients were allocated to the Mixed-HDF group if they had been treated for at least 6 months (24 weeks) with Post-HDF (baseline period) and subsequently for at least 1 year (48 weeks) with Mixed-HDF (observation period) (Fig 2). Patients were allocated to the Post-HDF group, if they had been treated for at least 1.5 years (24 baseline period + 48 weeks observation period) with Post-HDF. Thus, after the baseline period (“run-in phase”), the observation period compares the two stable prevalent patient groups: 1) patients who switched from Post-HDF to Mixed-HDF with 2) patients who remained on the Post-HDF treatment.

For inclusion into the analyses, at least 90% of treatments had to be performed during the entire observation period with the respective therapy. In order to have monthly information of the same patient population, only patients who were treated during the complete study period were included into the present study; thus, no drop-outs due to e.g. deaths, transplantations or center changes, occurred in the present study.

In an intraindividual analysis, we additionally compared the 6 months Post-HDF treatment (baseline period) of the Mixed-HDF group with the first 6 months of Mixed-HDF treatment (observation period) of these patients.

Prescription of dialysis modality was left at the physicians' discretion. The prescription of Mixed-HDF follows no distinct indication and patient characteristics.

### Outcome assessment

As all clinical parameters, the two primary outcomes, monthly ESA consumption and hemoglobin levels were routinely collected according to standardized clinical protocols and procedures of the NephroCare clinics [30].

All ESA doses were normalized to IUs (international units). For darbepoetin alfa (Aranesp, Amgen, Thousand Oaks, CA, USA) and methoxy-polyethylene-glycol-epoetin beta (MIRCERA; F. Hoffmann-La Roche Ltd, Basel, Switzerland), the original doses (in μg) were converted to IUs by using the factors 200 and 225, respectively [31, 32]. The monthly ESA dose was calculated as the total ESA consumption within a 4-week interval (28 days).

### Statistical analysis

#### Matching

Individual matching was performed to minimize differences between patients treated with Mixed-HDF and Post-HDF at baseline. Following baseline parameters considered for matching were a priori defined: dialysis center, gender, ESA consumption/4 weeks [categories: 0–10,000 IU, 10,000–30,000 IU, >30,000 IU], hemoglobin [categories: 0–10 g/dl, 10–11 g/dl, 11–12 g/dl, >12 g/dl], age, Charlson Comorbidity Index [CCI, categories: 1 (score≤2), 2 (score = 3), 3 (score = 4), 4 (score≥5)], body mass index (BMI) and vintage. For clinical parameters, the last assessment within the last three months of the baseline period was used.

In a first step, only those patient pairs were considered for matching who were treated in the same dialysis center and had the same gender. Secondly, we selected those patient pairs in the same category for ESA consumption and for hemoglobin. If no matched pairs could be found in this second step, patient pairs in the same ESA, but different hemoglobin categories–or subsequently vice versa–were selected. In case of multiple matches or no matching in these prior matching steps, manual matching was performed to select those patient pairs with the smallest differences in the additional variables age, CCI, BMI and vintage. This manual matching was performed by one person blinded for the longitudinal data.

#### Descriptive analyses

Descriptive analyses were performed for the two primary outcomes, monthly ESA consumption and hemoglobin levels as well as for operational dialysis parameters, treatment efficiency indexes and laboratory parameters (substitution volume, mean blood flow, effective treatment time, OCM (Online Clearance Monitoring) Kt/V, albumin, creatinine, beta-2-microglobulin, CRP, fluid overload, body weight, iron, ferritin, transferrin, TSAT (transferrin saturation), EPO resistance index, calcium, phosphate, parathyroid hormone, reticulocytes). EPO resistance index was calculated according to Marcelli et al [14], defined as EPO dose per body weight per hemoglobin level. OCM is integrated within Fresenius’ dialysis machines and allows to continuously measure the administered dialysis dose during each treatment based on conductivity variation during dialysis; the conductivity-based clearance, which reflects the clearance of electrolytes, is almost the same as the clearance of urea, as the diffusion coefficients of urea and electrolytes are almost equal [33]. For all clinical parameters, except for monthly iron and monthly ESA dose (see above), data from repeated assessments within 4 week intervals were averaged within patients, and these patient means were used for the calculation of descriptive statistics. Missing values were not replaced. Normally distributed variables are presented as mean ± SD; not normally distributed variables are summarized as median with 25th and 75th percentiles. Statistical analysis was performed with the paired t-test for normally distributed continuous variables, with the Wilcoxon Signed Rang test for not normally distributed continuous variables and with the McNemar test for dichotomous variables.

#### Inferential statistical analysis

Comparisons of Mixed-HDF *vs* Post-HDF regarding the courses of hemoglobin and monthly ESA consumption over the one-year observation period were based on the assumption of a linear trend over time. By estimating general linear mixed models with a *treatment x time* interaction term, we tested the two-sided hypothesis that the steepness of the linear slope of the respective parameter over the one-year observation period differs between the Mixed-HDF *vs* Post-HDF groups against the null hypothesis that the two slopes are parallel. Thus, we examined whether the rates of change in hemoglobin and monthly ESA consumption are different in patients on Mixed-HDF *vs* Post-HDF. In addition to the *treatment x time* interaction term, the main model included the dichotomous treatment variable (Mixed-HDF *vs* Post-HDF) and the continuous time variable (unit: 28 days) as fixed effects. Inter-correlations of measurements due to the matched pair design (i.e. correlations between patients belonging to the same pair) and repeated measurements of the respective outcome within patients over time (i.e. correlations of measurements obtained from the same patient) were accounted for by use of random effects for matched pairs and for patients nested within matched pairs. Thereby, to account for repeated measurements within patients in irregular time intervals, an autoregressive process of first order was applied as covariance structure. Thus, measurements obtained from the same patient shortly after each other were modeled to be stronger correlated than repeated measurements with longer in-between intervals. Based on the missing at random assumption, the models were estimated using all available assessments, thus not replacing any missing values. Due to the explorative nature of the study, no adjustment for multiple testing was performed.

Additionally, as we observed differences between Mixed- and Post-HDF patients in certain parameters over time in our descriptive analyses, we performed additional sensitivity analyses to adjust the estimation of the *treatment x time* interaction effects for repeated assessments of fluid overload, effective treatment time and mean blood flow during the one-year observation period. To this end, these three clinical parameters were included as additional fixed effects into the above-described linear mixed models. In the case of missing values on these three covariates, the last available value from up to three preceding months was imputed.

The statistical software SAS, version 9.4, was used for all analyses.

## Results

In total, 174 patients (87 patients on Mixed-HDF and 87 patients on Post-HDF) were included in the present analysis (Fig 1). 21 Mixed-HDF patients could not be matched to one of the Post-HDF patients. Baseline characteristics and operational treatment parameters of the final study population are presented in Table 1 and Table 2, respectively. Individual matching achieved a good balance between both treatment groups regarding the matching variables, except for dialysis vintage as patients receiving Post-HDF had a higher vintage than patients on Mixed-HDF. In both Post- and Mixed-HDF patients, diabetes mellitus was the most common cause for renal disease (S1 Table). Slightly more patients from the Post-HDF group had diabetes mellitus and peripheral vascular disease (S2 Table). No major differences were observed for other cardiovascular and hematological disorders between the two groups.

**Table 1:** Baseline characteristics of the study population.

	Total [n = 174]	Post-HDF [n = 87]	Mixed-HDF [n = 87]	Absolute difference	P
Age [years]	62.1±12.0	63.2±11.9	61.0±12.1	2.2	0.056
Gender [female]	54 [31.0%]	27 [31.0%]	27 [31.0%]	0	1.000
Charlson Comorbidity Index	3.0 [2;4]	3.0 [2;5]	3.0 [2;4]	0	0.115
Vintage [months]	55.5 [27;88]	59.0 [34;97]	48.0 [23;85]	11	0.023
BMI [kg/m^2^]	27.7±5.3	27.5±5.1	27.8±5.5	0.3	0.699
Hemoglobin [g/dl]	11.9±1.2	11.8±1.1	11.9±1.3	0.1	0.624
ESA/4 weeks [IU]	6000 [0;16000]	6000 [0;16000]	6000 [0;16000]	0	0.135
Patients without ESA	61 [35.1%]	31 [35.6%]	30 [34.5%]	1	0.763

Data are presented as mean ± SD, median [q1;q3] or n [%], as appropriate. For all parameters, except for monthly ESA dose, the last assessment of clinical data within the last three months of the baseline period was used. Statistical analysis was performed with the paired t-test for normally distributed continuous variables, with the Wilcoxon Signed Rang test for not normally distributed continuous variables and with the McNemar test for dichotomous variables.

**Table 2:** Treatment parameters of the two study groups at the baseline and during follow-up period.

Parameter	Baseline	Month 2	Month 4	Month 6	Month 8	Month 10	Month 12
**Mean blood flow** [ml/min]							
*Post-HDF*	383±41	383±44	383±40	382±40	380±42	379±41	380±43
*Mixed-HDF*	390±34	408±40	411±41	409±38	411±39	409±36	409±37
**Effective treatment time** [min]							
*Post-HDF*	237±18	238±18	238±18	238±17	238±17	238±17	238±18
*Mixed-HDF*	242±11	242±10	241±9	242±9	242±12	242±11	242±11
**OCM Kt/V**							
*Post-HDF*	1.79±0.37	1.81±0.39	1.82±0.39	1.85±0.41	1.80±0.38	1.81±0.40	1.83±0.39
*Mixed-HDF*	1.83±0.36	1.90±0.37	1.97±0.42	1.97±0.39	1.96±0.36	1.96±0.39	1.97±0.36
**Substitution volume** [l]							
*Post-HDF*	23.4±4.0	24.0±4.0	24.0±4.2	24.4±4.1	24.2±3.9	24.7±4.8	24.6±4.7
*Mixed-HDF*	23.6±3.6	38.9±5.0	38.6±5.4	38.1±4.9	38.0±5.0	37.7±4.6	38.0±4.7
**Intradialytic weight loss** [kg]							
*Post-HDF*	2.4±0.6	2.4±0.6	2.4±0.6	2.4±0.6	2.4±0.6	2.4±0.7	2.3±0.7
*Mixed-HDF*	2.5±0.7	2.5±0.6	2.6±0.7	2.6±0.6	2.6±0.6	2.5±0.6	2.5±0.6
**Fluid overload** [l]							
*Post-HDF*	1.4±1.3	1.5±1.6	1.5±1.4	1.4±1.3	1.3±1.6	1.2±1.7	1.6±1.4
*Mixed-HDF*	1.8±1.4	1.8±1.3	1.6±1.2	1.6±1.2	1.7±1.2	1.6±1.2	1.6±1.3

Data are presented as mean ± SD. For all parameters data from repeated assessments within 4 week intervals were averaged within patients.

Mean hemoglobin levels in the Post-HDF and Mixed-HDF groups were in the designated target range (11.8 *vs* 11.9 g/dl) and roughly two out of three patients received ESA (64.4 *vs* 65.5%) with a median dosage of 6000 IU/4 weeks in both groups. Of those 31 (Post-HDF) and 30 (Mixed-HDF) having received no ESA at baseline, 19 (Post-HDF) and 8 (Mixed-HDF) received at least once ESA during the following 12 months observation period. Monthly averages of Hb levels and total monthly ESA consumption of the total population are displayed in S3 and S4 Tables. Iron administration was comparable in both groups at baseline (iron dosage/4 weeks: 163 *vs* 166 mg). Used ESA and iron medications in this study are provided in S5 Table.

Upon switching from Post-HDF to Mixed-HDF, mean substitution volume increased according to the inherent characteristics of the mixed technique. In the Mixed-HDF group, effective treatment time was slightly higher and blood flow rate as well as Kt/V increased over time (Table 2 and S1 Fig). No clear differences in the clinical parameters albumin, creatinine, beta-2-microglobulin, calcium, phosphate and parathyroid hormone were observed in both groups over time, whereas mean CRP values were slightly higher but within the normal range in patients treated with Post-HDF compared to those on Mixed-HDF (Table 3 and S2 Fig). In line, when comparing the number of patients having CRP values above 10 mg/l during follow-up, we found that the mean number of patients having monthly CRP values above 10 mg/dl was higher in Post-HDF patients (6.7±3.5 patients/month) compared to Mixed-HDF patients (3.6±2.4). Moreover, reticulocytes slightly decreased in Mixed-HDF patients, while being stable in Post-HDF-patients (Table 3). Fluid overload decreased in patients from the Mixed-HDF group and reached the level of the Post-HDF group after 12 months. In contrast, the difference in intradialytic weight loss slightly increased over time between both groups with higher values in patients treated with Mixed-HDF (Table 2 and S3 Fig).

**Table 3:** Clinical parameters of the two study groups at the baseline and during follow-up.

Parameter	Baseline	Month 2	Month 4	Month 6	Month 8	Month 10	Month 12
**β2-M** [mg/l]							
*Post-HDF*	24.3±8.0	20.2±8.5	28.5±10.1	24.9±7.0	28.3±8.1	27.8±9.9	25.7±7.7
*Mixed-HDF*	17.3±10.4	23.9±6.9	17.4±9.6	22.0±8.8	26.4±16.9	21.1±6.9	22.8±9.3
**Albumin** [g/dl]							
*Post-HDF*	3.9±0.3	3.8±0.5	3.8±0.4	3.8±0.4	3.7±0.4	3.9±0.5	3.8±0.5
*Mixed-HDF*	3.8±0.4	3.8±0.3	3.8±0.3	3.9±0.3	4.0±0.3	3.9±0.3	3.9±0.3
**Creatinine** [mg/dl]							
*Post-HDF*	8.4±2.2	8.4±2.1	8.3±2.0	8.4±2.2	8.1±2.0	8.6±2.3	8.3±2.2
*Mixed-HDF*	8.9±1.9	8.9±1.9	8.5±2.0	8.7±1.9	8.9±2.2	8.6±2.0	9.0±2.2
**CRP** [mg/l]							
*Post-HDF*	2.7 [1.3;7.1]	5.0 [1.6;8.7]	6.5 [2.6;10.8]	4.2 [1.5;14.2]	5.8 [2.6;8.8]	7.4 [2.6;9.7]	5.0 [1.7;11.8]
*Mixed-HDF*	1.0 [0.6;3.2]	2.8 [1.0;4.3]	2.4 [1.0;8.3]	1.9 [0.7;5.0]	2.0 [0.9;5.6]	1.5 [0.3;4.0]	3.6 [1.2;7.1]
**Ferritin** [μg/l]							
*Post-HDF*	496±369	441±303	337±210	395±226	386±364	409±321	479±358
*Mixed-HDF*	349±228	522±341	486±385	438±227	397±310	493±297	570±362
**Transferrin** [mg/dl]							
*Post-HDF*	199±28	218±56	189±52	200±54	193±45	187±49	197±59
*Mixed-HDF*	205±32	175±28	193±47	187±35	191±45	184±48	184±45
**TSAT** [%]							
*Post-HDF*	27±10	26±11	26±14	27±14	26±16	27±12	24±10
*Mixed-HDF*	25±7	38±17	29±10	32±15	30±15	30±15	32±14
**Iron dose** [mg/4weeks]							
*Post-HDF*	163±170	186±187	168±192	162±184	173±183	154±176	156±179
*Mixed-HDF*	166±180	155±153	147±165	127±154	125±141	144±161	135±141
**EPO resistance index** [IU/kg/(g/dl)]							
*Post-HDF*	1.56 [0;5.34]	2.14 [0;5.44]	2.02 [0;6.08]	2.34 [0;6.53]	2.53 [0;9.33]	1.44 [0;5.54]	2.15 [0;6.91]
*Mixed-HDF*	2.23 [0;5.76]	1.99 [0;5.80]	1.65 [0;6.08]	0.67 [0;4.04]	1.78 [0;5.29]	1.60 [0;4.01]	0.69 [0;3.55]
**Calcium** [mg/dl]							
*Post-HDF*	9.3±0.8	9.2±0.7	9.1±0.6	9.1±0.6	9.1±0.6	9.0±0.6	9.1±0.7
*Mixed-HDF*	9.1±0.8	9.1±0.7	9.2±0.8	9.1±0.6	9.2±0.7	9.1±0.6	9.1±0.6
**Phosphate** [mg/dl]							
*Post-HDF*	4.4±1.5	4.6±1.6	4.6±1.5	4.6±1.4	4.6±1.6	4.7±1.9	4.4±1.7
*Mixed-HDF*	4.3±1.2	4.4±1.1	4.3±1.1	4.3±1.2	4.3±1.0	4.3±1.1	4.3±1.1
**Parathyroid hormone** [pg/ml]							
*Post-HDF*	226.2 [89.2;456.0]	217.7 [138.2;303.8]	390.8 [166.7;681.0]	251.4 [171.4;379.0]	333.0 [212.0;497.0]	237.0 [175.0;317.9]	193.1 [107.0;391.0]
*Mixed-HDF*	220.5 [174.0;345.5]	195.5 [138.3;382.8]	214.1 [126.4;335.0]	196.0 [138.0;320.5]	272.1 [131.0;486.5]	230.0 [149.0;367.0]	194.3 [149.0;325.4]
**Reticulocytes** [%]							
*Post-HDF*	2.1±1.6	2.1±1.4	2.3±1.4	1.7±0.7	2.1±1.8	1.7±0.9	2.2±1.7
*Mixed-HDF*	2.1±1.2	2.1±1.1	1.8±0.9	1.4±0.7	1.9±0.7	1.5±0.5	1.6±0.5

Data are presented as mean ± SD or median [q1;q3], as appropriate. For all parameters, except for iron dose, data from repeated assessments within 4 week intervals were averaged within patients.

**Fig 3:**
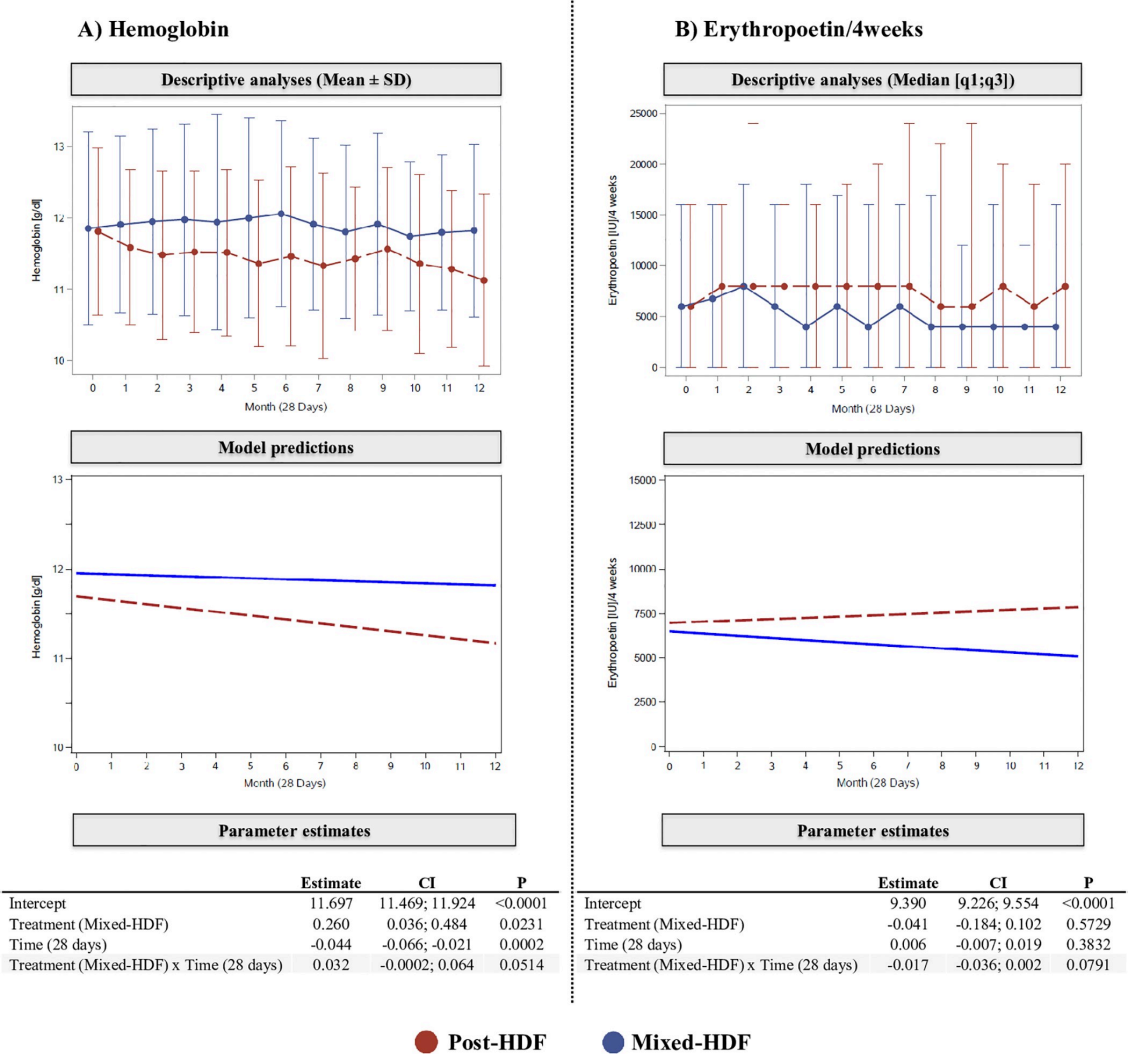
**Descriptive analyses (upper part) and inference statistical models (middle and lower part) for hemoglobin (g/dl) and erythropoietin consumption (ESA/4weeks, IU).** In descriptive analyses data are presented as mean ± SD for hemoglobin and as median with 25^th^ and 75^th^ percentiles [q1;q3] for ESA consumption. In inference statistical models we calculated the *treatment x time* interaction term to test for both parameters (hemoglobin and ESA/4 weeks) whether the steepness of the slopes differ between both groups. Mixed-HDF: Mixed-dilution hemodiafiltration, Post-HDF: Post-dilution hemodiafiltration.

Regarding anemia parameters, patients treated with Mixed-HDF showed stable hemoglobin levels throughout the observation period (11.9±1.3 g/dl at baseline, 11.8±1.2 g/dl at month 12), while patients treated with Post-HDF experienced a slight decrease in hemoglobin levels over time (11.8±1.1 g/dl at baseline, 11.1±1.2 g/dl at month 12) (Fig 3A). This difference in course over time between the two treatment groups was also supported by testing the *treatment x time* interaction term of our inference statistical model, which, however failed to achieve statistical significance (p = 0.0514). Accordingly, patients treated with Mixed-HDF received less ESA than patients treated with Post-HDF during the complete observation period (Fig 3B). In the prediction model, the difference in the course of ESA administration over time between both groups however also failed to attain statistical significance (*treatment x time*: p = 0.0791). These results were confirmed by the intraindividual analysis comparing the 6 months Post-HDF treatment with the first 6 months of Mixed-HDF treatment of the Mixed-HDF group (*treatment x time*: Hb: p = 0.1146; ESA: p = 0.0025; S4 Fig).

In line with these observations, the EPO resistance index was numerically lower in the Mixed-HDF group during the observation period (Table 3). Iron administration as well as ferritin and transferrin had a comparable course over time in both groups (Table 3 and S5 Fig). TSAT was slightly higher in Mixed-HDF patients as compared to Post-HDF patients.

Finally, we additionally performed sensitivity analyses given the observed differences in fluid overload, mean blood flow and effective treatment time in our descriptive analyses. Adjustment for these parameters strengthened our previous observations (hemoglobin: p = 0.0124; ESA: p = 0.0687 for the *treatment x time* interaction estimate) (Table 4).

**Table 4:** Sensitivity analyses: Inference statistics for comparison between Mixed- and Post-HDF with adjustment for overhydration, mean blood flow and effective treatment time.

		Estimate	CI	P
Hemoglobin [g/dl]	Intercept	12.224	11.214; 13.233	<0.0001
	Treatment (Mixed-HDF)	0.313	0.078; 0.548	0.0090
	Time (28 days)	-0.052	-0.076; -0.028	<0.0001
	Treatment (Mixed-HDF) x Time (28 days)	0.042	0.009; 0.076	0.0124
	Fluid overload [l]	-0.072	-0.111; -0.033	0.0003
	Effective treatment time [min]	0.003	-0.001; 0.006	0.0982
	Mean blood flow [ml/min]	-0.003	-0.004; -0.002	<0.0001
log(epo+5000)	Intercept	9.695	8.679; 10.711	<0.0001
	Treatment (Mixed-HDF)	-0.024	-0.176; 0.128	0.7554
	Time (28 days)	0.009	-0.005; 0.023	0.2167
	Treatment (Mixed-HDF) x Time (28 days)	-0.018	-0.038; 0.001	0.0687
	Fluid overload [l]	0.008	-0.013; 0.030	0.4522
	Effective treatment time [min]	-0.0003	-0.004; 0.003	0.8780
	Mean blood flow [ml/min]	-0.001	-0.002; 0.001	0.3656

NB: unadjusted results of main model are presented in Fig 3. The monthly ESA dose (epo) was highly skewed to the right, which necessitated log-transformation prior to modeling. Since there were zero values and to better approach normality, a constant of 5000 was added prior to log-transformation of this variable. Model estimation was thus performed on the log(x+5000) scale. CI: confidence interval

## Discussion

In this multicenter, retrospective cohort study we compared the effects of Mixed-HDF versus Post-HDF on anemia management. Our hypothesis-generating results suggest that patients treated with Mixed-HDF may have clinical benefits in terms of hemoglobin concentration and ESA consumption compared to those patients treated with the traditional Post-HDF.

Anemia is a major comorbidity in patients with ESRD, associated with poor quality of life and increased mortality [24]. Besides reduced EPO production, other factors may contribute to anemia in CKD, including reduced erythrocyte life-span, hyperparathyroidism, inhibition of erythropoiesis by uremic toxins, iron metabolism disorders and impaired dietary iron and vitamin absorption [24].

ESA therapy is a central component in the treatment of anemia in CKD. According to DOPPS data a significant rise in ESA use and hemoglobin levels occurred during the period 1996–2008 [34]. However, together with the impressive benefits of ESAs on anemia management, increasing evidence has alerted the community to the risk of important negative outcomes as a consequence of high ESA doses, especially in terms of cardiovascular disease and cancer incidence in dialysis patients [24–29]. This has promoted a more cautious approach to ESA administration, leading to a worldwide decrease in ESA doses and target hemoglobin levels over recent years [35].

New strategies for the management of anemia, such as hypoxia-inducible transcription factors (HIF) stabilizers, or hepcidin inhibitors/antagonists might have an important role in the near future [35, 36]. In this context, also optimized dialysis modalities, especially HDF, have gained substantial interest as several studies suggest clinical benefits of HDF over conventional HD in terms of ESA / ERI reduction and better anemia control [6, 11–15]. However, data in this context is conflicting, as other studies could not confirm these results [1, 16–18]. These discrepant findings may be explained by the fact that these previous studies did not differentiate the substitution mode of HDF and did not adjust convection volume to match postdilution volume when comparing the two treatment modalities in the context of anemia management. Furthermore, these studies did not consider additional confounding factors such as fluid overload or inflammation.

Results of our present exploratory study might provide a first piece of evidence that the substitution mode in Mixed-HDF may have a beneficial effect on anemia. We observed that Mixed-HDF patients maintained stable hemoglobin values with lower ESA doses compared to Post-HDF patients. Post-HDF patients experienced a slight while not significant decrease of hemoglobin values even with higher ESA consumption and without substantial differences in iron metabolism parameters and iron administration. Interestingly, the difference in hemoglobin values becomes apparent already after 1–2 months of treatment, while ESA consumption follows this difference 1–2 months later. Differences in Hb trends and ESA consumption between the two treatment groups failed to reach statistical significance, which was however partly revised after adjusting for additional factors in sensitivity analyses. Possibly, the limited sample size and the large variation in haemoglobin and ESA levels among patients are potential factors influencing statistical significance levels. Notwithstanding, our findings appear to be of clinical relevance, given that the monthly median ESA consumption of patients on Mixed-HDF at the end of the observation period was 50% lower than those of patients on Post-HDF. Notably, due to the observational nature of the present study, our results have to be interpreted carefully. Additional evidence is warranted to confirm our findings and evaluate the modality also in the context of its potential economic impact.

We can only speculate about potential reasons for the results observed in the present study. Efficient removal of middle and large size uremic toxins, which may contribute to impaired erythropoiesis in dialysis patients, may be one advantage of Mixed-HDF in anemia management. Indeed, better elimination of middle and large size molecules has been linked with reduced ESA doses [6, 11–15]. Moreover, Maduell et al [37] reported a significant improvement of anemia, when the substitution rate was substantially increased which itself could be linked to a better removal of uremic toxins. The high convective volume achieved in Mixed HDF might be in line with this observation. One important metabolite in this context is hepcidin: its excess is considered a major contributor of the disorders in iron homeostasis and anemia in CKD patients [24]. Recent studies demonstrated that HDF removes hepcidin more efficiently than conventional HD, which may explain the observed clinical benefits concerning anemia in HDF-treated patients [15, 38]. Whether Mixed-HDF can eliminate hepcidin more efficiently than Post-HDF needs further investigation. Removal of proinflammatory cytokines also plays an important role in anemia. Inflammatory cytokines can impair erythropoiesis and may be, in part, responsible for ESA resistance in CKD [39–41]. Interestingly, several studies have shown that HDF has the potential to reduce inflammation [8–10]. As inflammatory cytokines were not assessed in our study, we cannot link our results with this hypothesis. However, patients treated with Mixed-HDF had slightly lower CRP values than patients treated with Post-HDF. Finally, the most striking difference between the two substitution techniques is the hemo-rheological and hydraulic conditions within the dialyzers during treatments. The feedback mechanism of Mixed-HDF prevents potential hemoconcentration and hyper-viscosity by infusing substitution fluid in pre-dilution mode when high hydraulic pressure regimen establishes within the dialyzer, irrespective of patient- and technical conditions of the treatment. This may prevent sub-clinical (micro)hemolysis, which is usually undetected as it falls below any acute hemolytic threshold but may indeed result in increased demand for ESA. The observed difference in reticulocyte values between the two groups may indicate reduced hemolysis in Mixed-HDF patients. This, together with some direct volume effects, may also partly explain the fast changes in haemoglobin values observed between the two treatment groups. However, as this study was not designed to explore this aspect in detail further research on hemodynamics and rheology is certainly needed to confirm our hypothesis.

Our study has certain limitations. First, the observational and explorative design of our study does not allow to draw any conclusions regarding the causality of our results. Second, the limited number of patients possibly prevented more stringent results. On the other hand, implementation of Mixed-HDF is still sparse in dialysis clinics, and only a restricted number of patients is treated with this technique. Notably, this is the largest comparative analysis with patients on Mixed-HDF up to now. Additionally, some parameters–such as reticulocytes–are not evaluated monthly in the routine dialysis practise, which subsequently leads to missing values during follow-up. However, missing values were evenly distributed among the two treatment groups. Additionally, we did not have data regarding treatment side effects, such as clotting or vascular access problems, and no information regarding blood transfusions. Moreover, although we carried out matching very thoroughly, certain parameters differed between Mixed- and Post-HDF patients at baseline, such as vintage, treatment time, ferritin or fluid overload. However, our sensitivity analyses, adjusting for distinct parameters, strengthened our results. Finally, we focussed in the present analysis on patients who were treated three times the week and who had fistula/graft as vascular access, in order to reduce the heterogeneity of our study population; this limits the generalisation of our present results and further studies including patients with different treatment characteristics are clearly needed. Notwithstanding, our explorative study provides a first indication that distinct treatment modalities may positively impact anemia management in dialysis patients. The potential clinical and economical relevance of this topic may stimulate further research to verify our observations.

In summary, hypothesis-generating results of our study suggest that patients treated with Mixed-HDF may have clinical benefits in terms of anemia management. These findings may also have economic consequences given the high economic burden of ESA therapy in the treatment of dialysis patients. Future studies are needed to confirm our results and to provide additional evidence on the potential beneficial effects of Mixed-HDF.

## Supporting information

S1 Fig Descriptive analyses for the dialysis performance parameters: Substitution volume [l], mean blood flow [ml/min], effective treatment time [min] and OCM Kt/V. Data are presented as mean ± SD for all parameters. Mixed-HDF: Mixed-dilution hemodiafiltration, Post-HDF: Post-dilution hemodiafiltration.
(PDF)

S2 Fig Descriptive analyses for the laboratory parameters: Albumin [g/dl], creatinine [mg/dl], Beta-2-microglobulin [mg/l] and CRP [mg/l]. Data are presented as mean ± SD for albumin, creatinine and Beta-2-microglobulin and as median with 25^th^ and 75^th^ percentiles [q1;q3] for CRP. CRP: C-reactive protein, Mixed-HDF: Mixed-dilution hemodiafiltration, Post-HDF: Post-dilution hemodiafiltration.
(PDF)

S3 Fig Descriptive analysis for fluid overload [l] and delta body weight [kg]. Data are presented as mean ± SD for both parameters. Mixed-HDF: Mixed-dilution hemodiafiltration, Post-HDF: Post-dilution hemodiafiltration.
(PDF)

S4 Fig **Intraindividual analysis: inference statistical models for A) hemoglobin (g/dl) and B) erythropoietin consumption (ESA/4weeks, IU).** *Treatment x time* interaction terms were calculated to test for both parameters (hemoglobin and ESA/4 weeks) whether the steepness of the slopes differ between the baseline period (Post-HDF) of the Mixed-HDF group and the first 6 months of the observation period (Mixed-HDF). Mixed-HDF: Mixed-dilution hemodiafiltration, Post-HDF: Post-dilution hemodiafiltration.
(PDF)

S5 Fig Descriptive analysis for the iron metabolism parameters: Iron/4weeks [mg], ferritin [μg/l], transferrin [mg/dl] and TSAT [%]. Data are presented as mean ± SD for all parameters. Mixed-HDF: Mixed-dilution hemodiafiltration, Post-HDF: Post-dilution hemodiafiltration.
(PDF)

S1 Table Cause of renal disease of the study patients. (PDF)

S2 Table Major cardiovascular and hematological comorbidities of the study patients. (PDF)

S3 Table Monthly averages of hemoglobin levels [g/dl] of the study patients within the study period. (PDF)

S4 Table Total monthly ESA consumption [IU] of the study patients within the study period. (PDF)

S5 Table Used ESA and iron medications in the study. (PDF)
