# Supplementary figures and images for: Effects of high-volume online mixed-hemodiafiltration on anemia management in dialysis patients
Source: PLoS One. 2019 Feb 22;14(2):e0212795. doi: 10.1371/journal.pone.0212795 (PMC6386285; doi:10.1371/journal.pone.0212795)

**A) Substitution volume**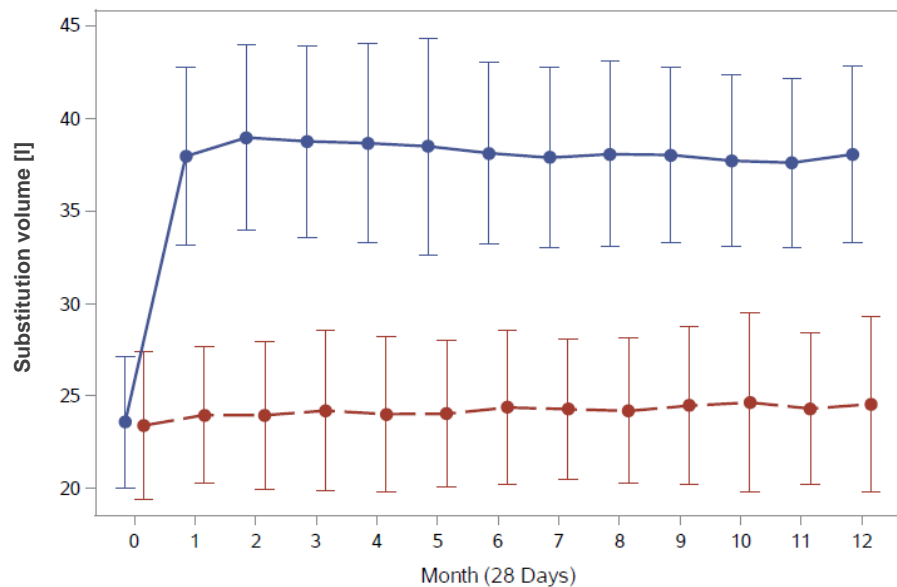**B) Mean blood flow**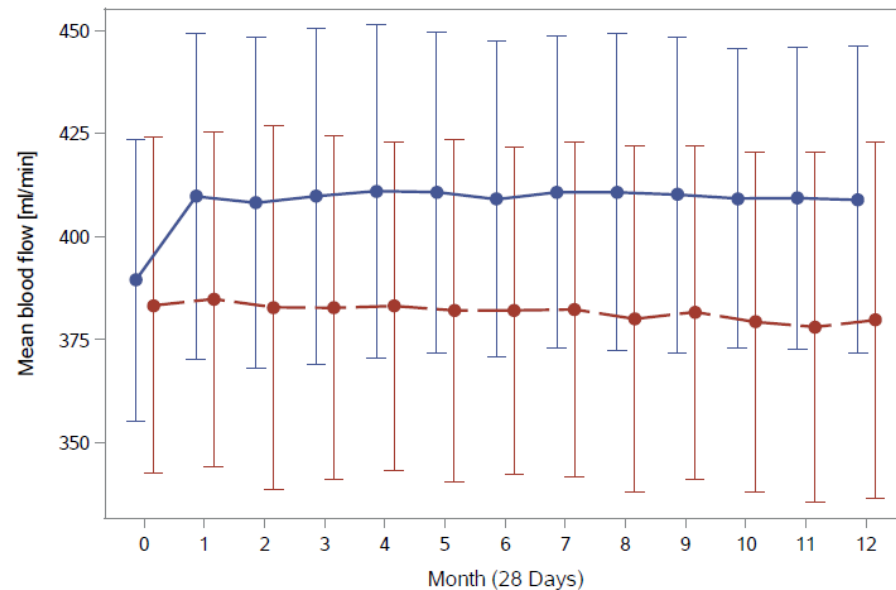**C) Effective treatment time**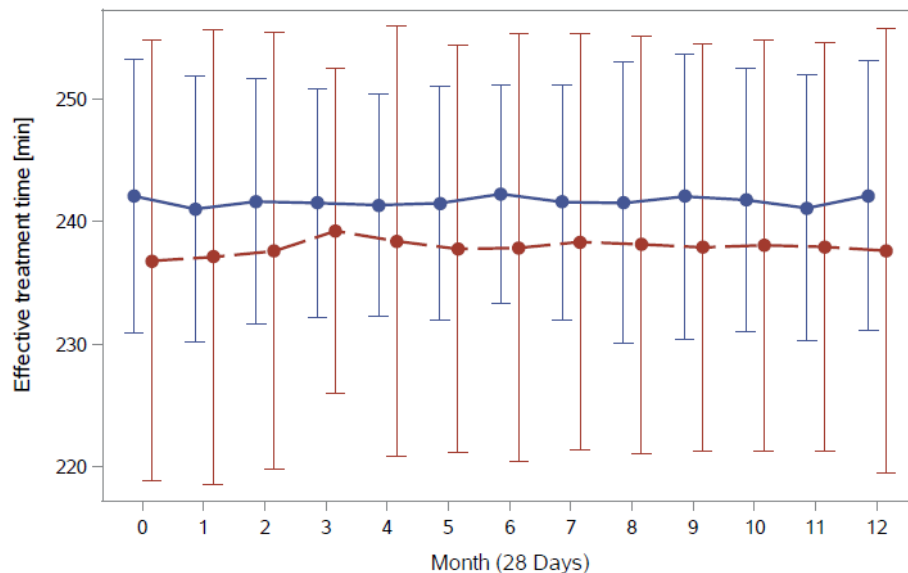**D) OCM Kt/V**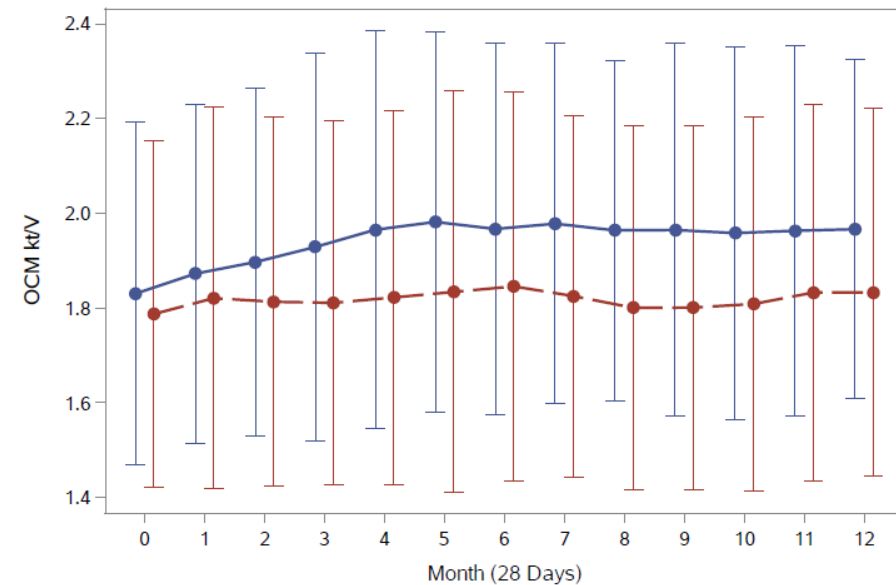

● Post-HDF

● Mixed-HDF

Supplement: S1 Fig — Data are presented as mean ± SD for all parameters. Mixed-HDF: Mixed-dilution hemodiafiltration, Post-HDF: Post-dilution hemodiafiltration. (PDF) [file pone.0212795.s001.pdf]

**A) Albumin**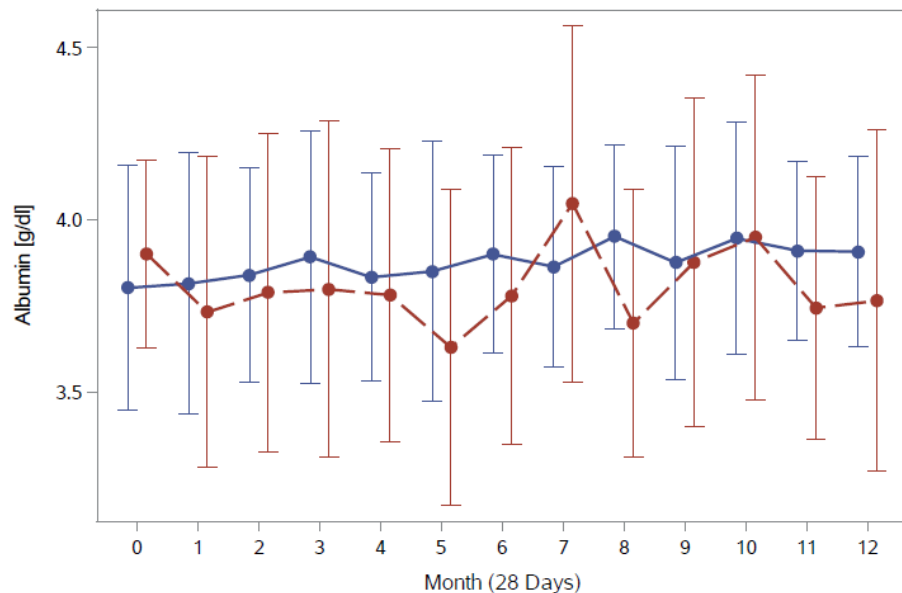**B) Creatinine**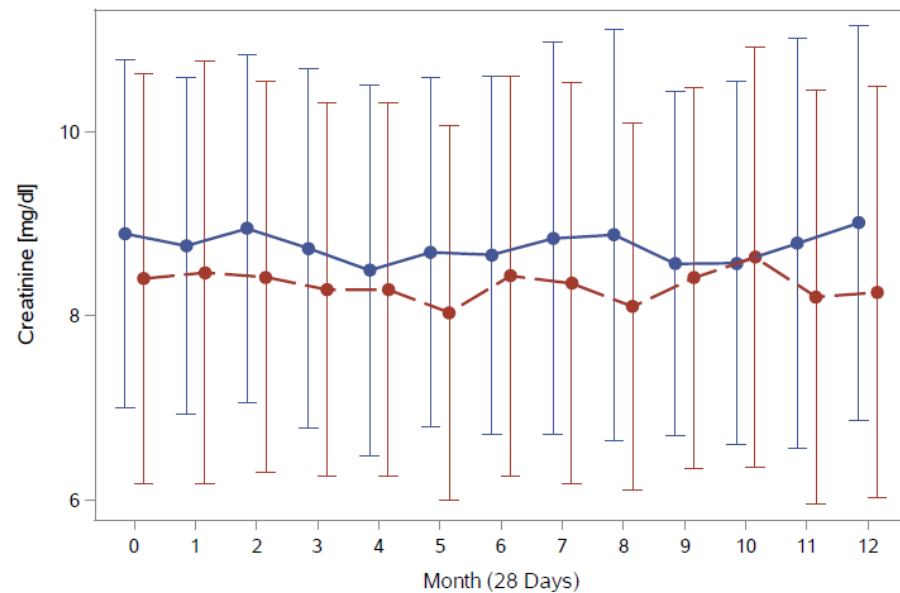**C) Beta-2-microglobulin**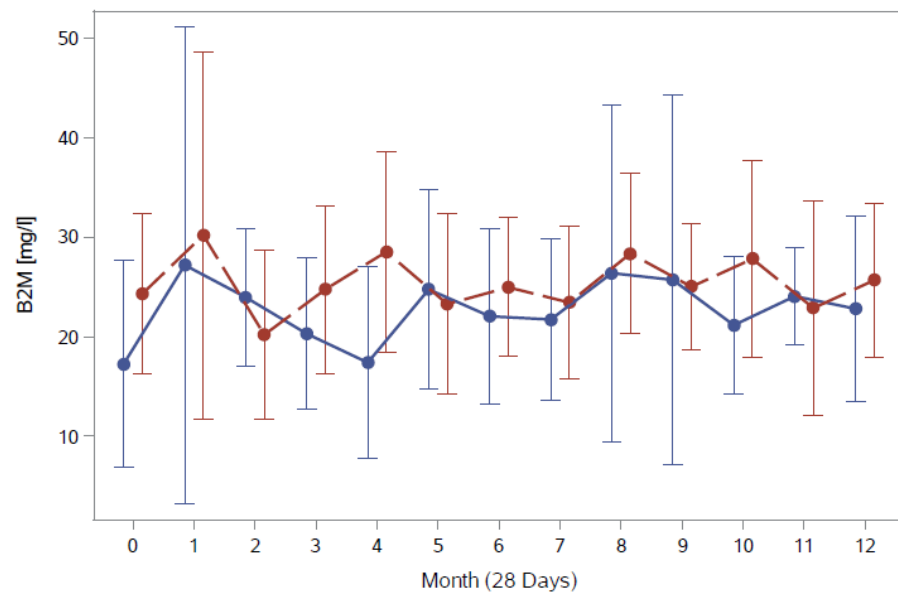**D) CRP**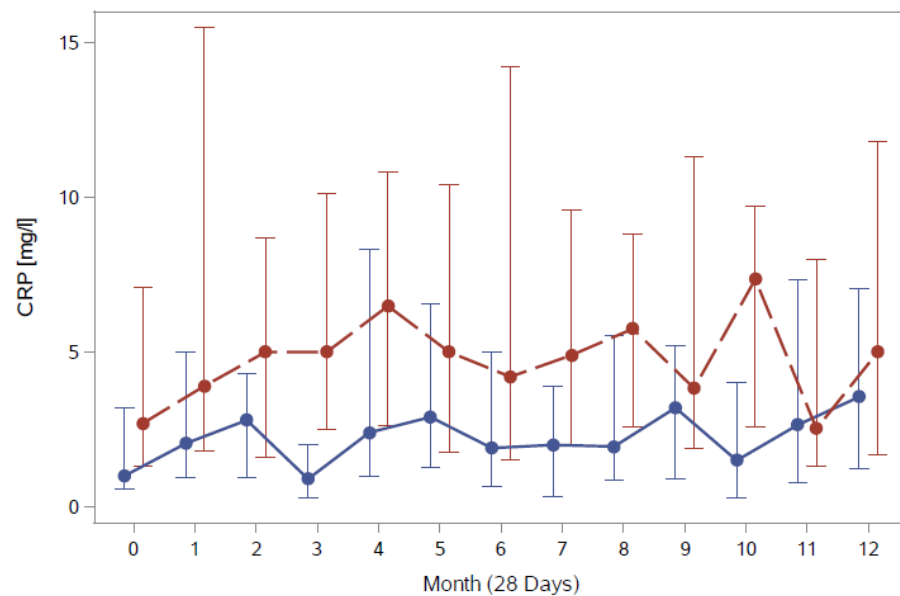

● Post-HDF

● Mixed-HDF

**S2 Fig**

Supplement: S2 Fig — Data are presented as mean ± SD for albumin, creatinine and Beta-2-microglobulin and as median with 25th and 75th percentiles [q1;q3] for CRP. CRP: C-reactive protein, Mixed-HDF: Mixed-dilution hemodiafiltration, Post-HDF: Post-dilution hemodiafiltration. (PDF) [file pone.0212795.s002.pdf]

**A) Fluid overload**

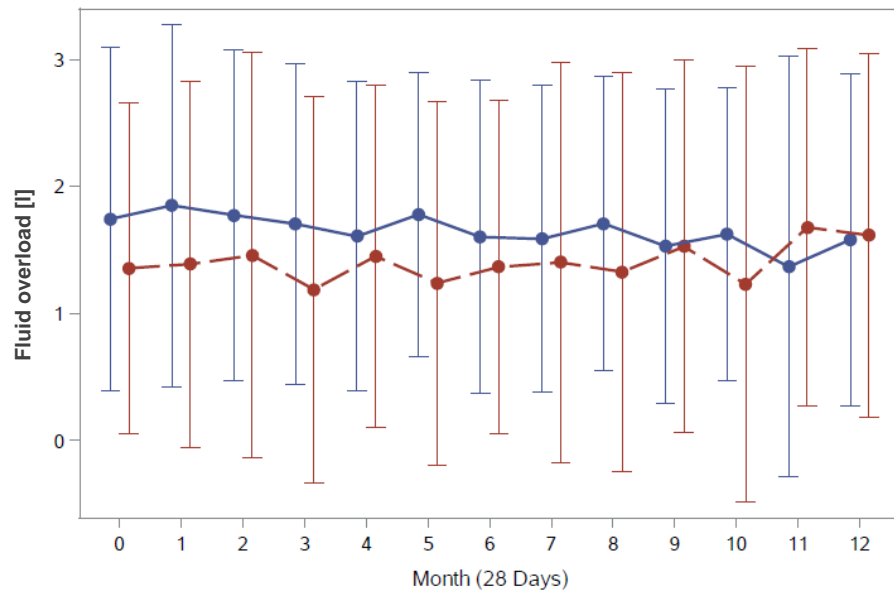

**B) Intradialytic weight loss**

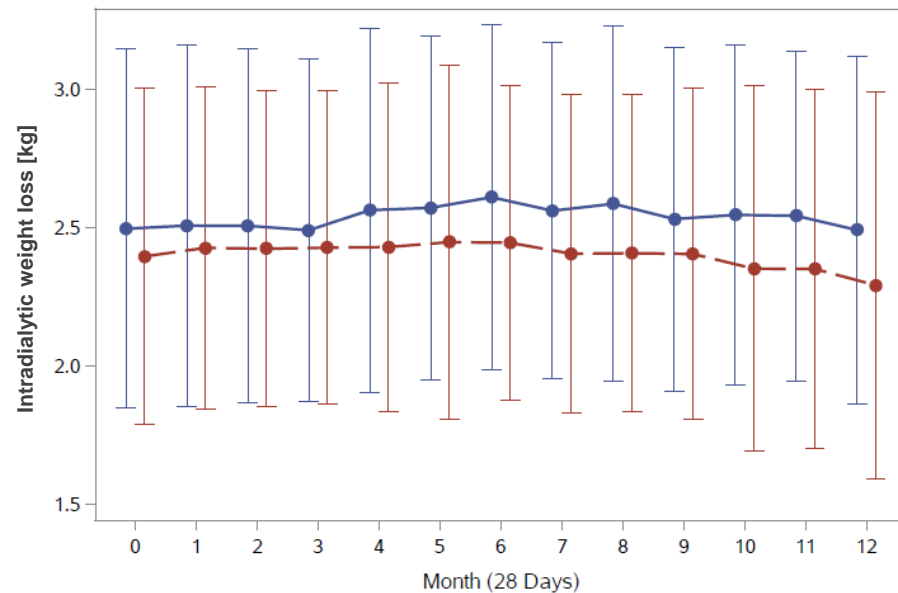

● **Post-HDF**

● **Mixed-HDF**

**S3 Fig**

Supplement: S3 Fig — Data are presented as mean ± SD for both parameters. Mixed-HDF: Mixed-dilution hemodiafiltration, Post-HDF: Post-dilution hemodiafiltration. (PDF) [file pone.0212795.s003.pdf]

## A) Hemoglobin

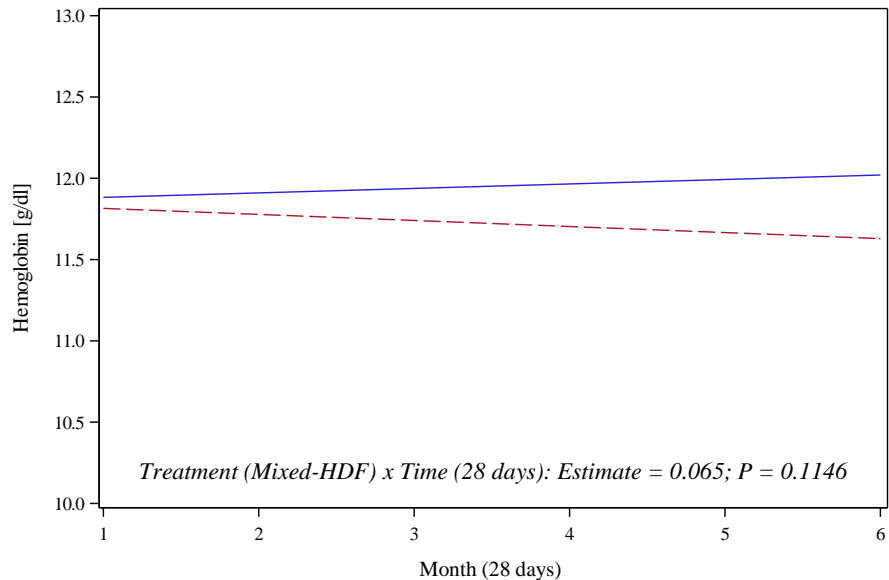

## B) Erythropoietin/4weeks

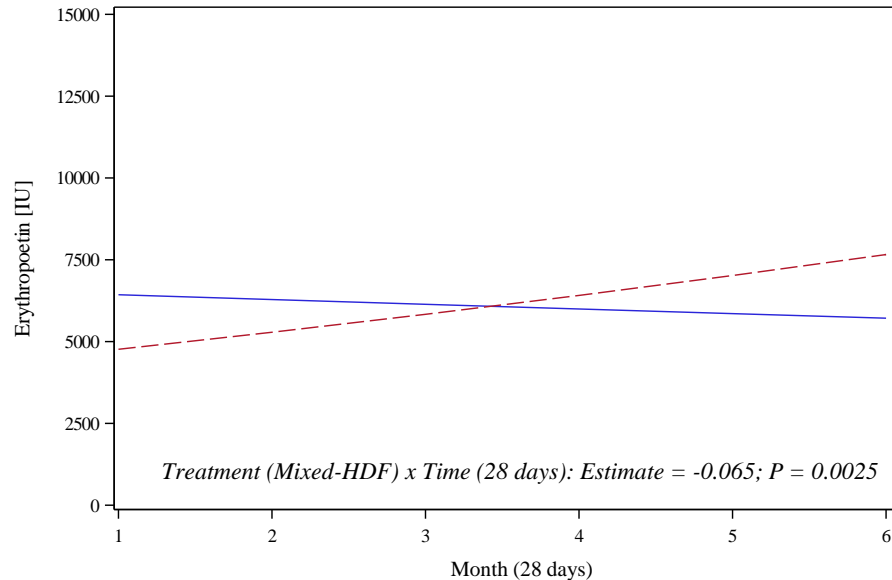

● **Post-HDF**

● **Mixed-HDF**

**S4 Fig**

Supplement: S4 Fig — Intraindividual analysis: inference statistical models for A) hemoglobin (g/dl) and B) erythropoietin consumption (ESA/4weeks, IU). Treatment x time interaction terms were calculated to test for both parameters (hemoglobin and ESA/4 weeks) whether the steepness of the slopes differ between the baseline period (Post-HDF) of the Mixed-HDF group and the first 6 months of the observation period (Mixed-HDF). Mixed-HDF: Mixed-dilution hemodiafiltration, Post-HDF: Post-dilution hemodiafiltration. (PDF) [file pone.0212795.s004.pdf]

**A) Iron/4weeks**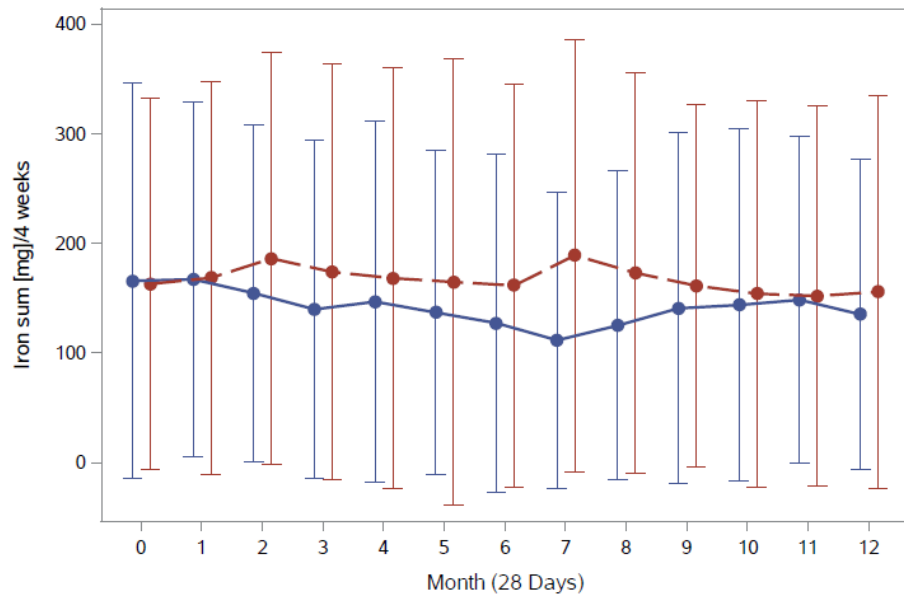**B) Ferritin**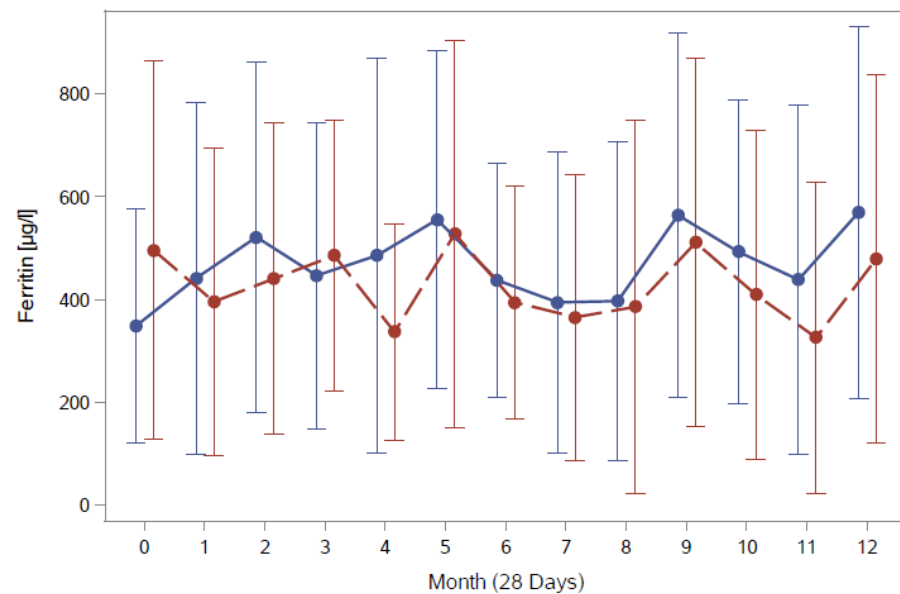**C) Transferrin**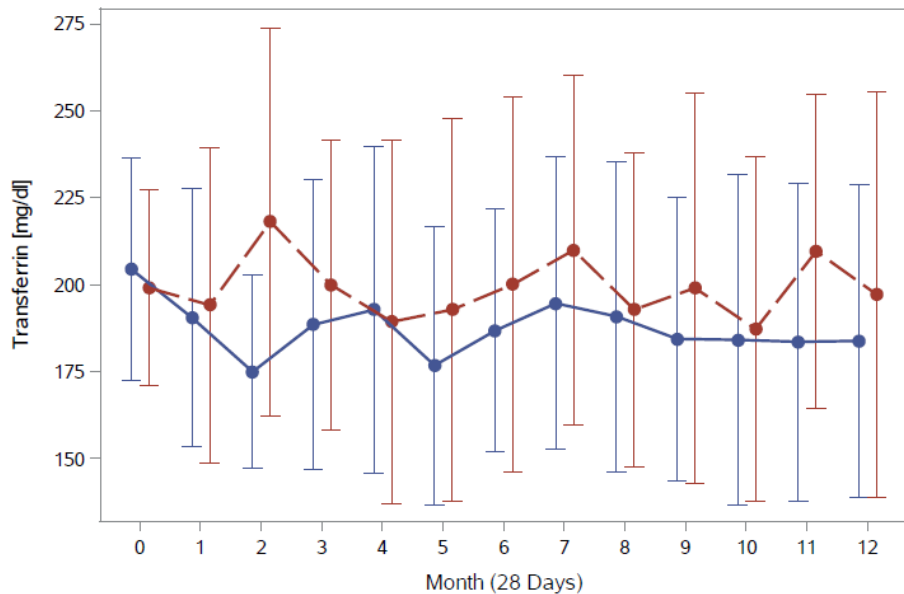**D) TSAT**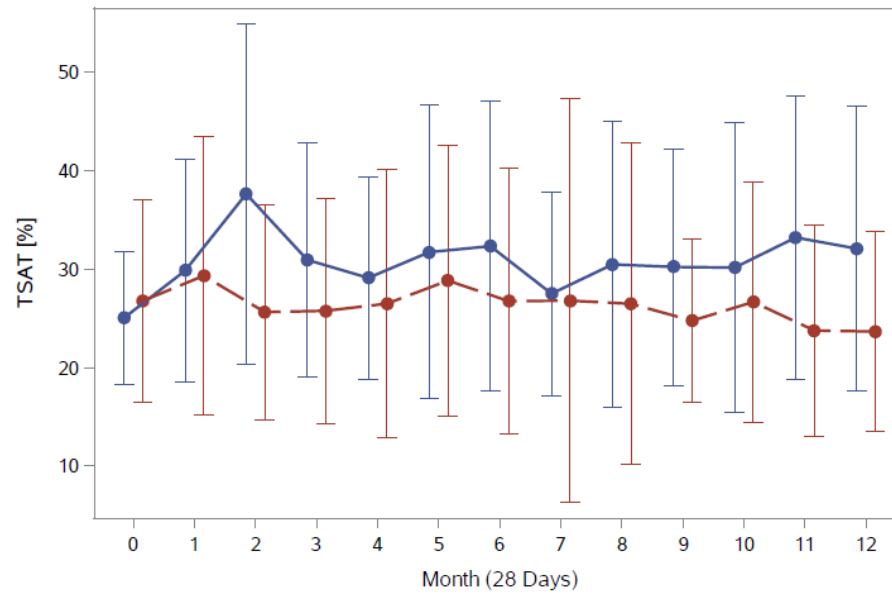

● Post-HDF

● Mixed-HDF

**S5 Fig**

Supplement: S5 Fig — Data are presented as mean ± SD for all parameters. Mixed-HDF: Mixed-dilution hemodiafiltration, Post-HDF: Post-dilution hemodiafiltration. (PDF) [file pone.0212795.s005.pdf]
