# Supplementary material for: Effects of high-volume online mixed-hemodiafiltration on anemia management in dialysis patients
Source: PLoS One. 2019 Feb 22;14(2):e0212795. doi: 10.1371/journal.pone.0212795 (PMC6386285; doi:10.1371/journal.pone.0212795)
Supplement: S1 Table — (PDF) [file pone.0212795.s006.pdf]

S1 Table. Cause of renal disease of the study patients.

	**Total [n = 174]**	**Post-HDF [n = 87]**	**Mixed-HDF [n = 87]**
Diabetes mellitus	31 [17.8%]	17 [19.5%]	14 [16.1%]
Hypertension	22 [12.6%]	9 [10.3%]	13 [14.9%]
Glomerular disease	21 [12.1%]	8 [9.2%]	13 [14.9%]
Congenital malformation	21 [12.1%]	10 [11.5%]	11 [12.6%]
Renal tubulo-interstitial disease	8 [4.6%]	1 [1.1%]	7 [8.1%]
Amyloidosis	3 [1.7%]	2 [2.3%]	1 [1.1%]
Cancer	3 [1.7%]	1 [1.1%]	2 [2.3]
Other	7 [4.0%]	4 [4.6%]	3 [3.4%]
Unknown	58 [33.3%]	35 [40.2%]	23 [26.4%]
