# Supplementary material for: Effects of high-volume online mixed-hemodiafiltration on anemia management in dialysis patients
Source: PLoS One. 2019 Feb 22;14(2):e0212795. doi: 10.1371/journal.pone.0212795 (PMC6386285; doi:10.1371/journal.pone.0212795)
Supplement: S2 Table — (PDF) [file pone.0212795.s007.pdf]

S2 Table. Major cardiovascular and hematological comorbidities of the study patients.

	**Total [n = 174]**	**Post-HDF [n = 87]**	**Mixed-HDF [n = 87]**
Myocardial infarction	8 [4.6%]	6 [6.9%]	2 [2.3%]
Congestive heart failure	19 [10.9%]	9 [10.3%]	10 [11.5%]
Peripheral vascular disease	26 [14.9%]	20 [23.0%]	6 [6.9%]
Cerebrovascular disease	12 [6.9%]	7 [8.1%]	5 [5.7%]
Diabetes mellitus	42 [24.1%]	24 [27.6%]	18 [20.7%]
Hemolytic anemias	0 [0%]	0 [0%]	0 [0%]
Thalassemia	0 [0%]	0 [0%]	0 [0%]
Sickle cell disorders	0 [0%]	0 [0%]	0 [0%]
Aplastic anemias	1 [0.6%]	1 [1.1%]	0 [0%]
Coagulation defects	1 [0.6%]	0 [0%]	1 [1.1%]
Hemorrhagic conditions	2 [1.1%]	0 [0%]	2 [2.3%]
