# Supplementary material for: Effects of high-volume online mixed-hemodiafiltration on anemia management in dialysis patients
Source: PLoS One. 2019 Feb 22;14(2):e0212795. doi: 10.1371/journal.pone.0212795 (PMC6386285; doi:10.1371/journal.pone.0212795)
Supplement: S3 Table — (PDF) [file pone.0212795.s008.pdf]

S3 Table. Monthly averages of hemoglobin levels [g/dl] of the study patients within the study period.

Patient	Baseline	Month 1	Month 2	Month 3	Month 4	Month 5	Month 6	Month 7	Month 8	Month 9	Month 10	Month 11	Month 12
Mixed-HDF													
1	11.9	10.3	10.9	10.3	11.1	11.7	10.8	10.2	10.6	10.7	11.5	11.7	11.1
2	12.5	12.7	13.0	12.7	12.7	11.7	11.7	11.6	11.9	11.8	11.6	11.3	11.4
3	12.6	11.4	12.4	13.1	10.6	11.2	12.0	11.3	10.8	11.4	12.2	NA	12.4
4	12.3	12.3	13.0	NA	12.0	11.0	11.2	11.0	11.4	12.2	11.6	12.3	11.3
5	10.7	11.5	11.6	11.7	11.4	11.7	NA	11.3	11.7	12.0	10.9	11.3	11.2
6	11.7	12.4	12.2	NA	11.6	12.3	12.0	12.2	11.0	11.2	11.7	12.4	12.0
7	13.8	14.5	16.0	16.0	17.5	15.3	14.8	14.6	13.8	13.7	NA	13.3	13.8
8	11.3	NA	10.0	10.8	11.0	10.9	10.6	NA	11.3	11.0	11.1	11.4	10.7
9	13.6	14.2	15.2	15.7	16.0	15.9	15.3	14.2	NA	14.3	13.8	14.0	15.0
10	10.8	10.9	11.6	10.5	10.3	11.3	10.6	10.0	10.4	10.8	10.9	11.0	11.1
11	14.0	12.1	11.3	NA	9.1	9.9	11.4	11.6	NA	11.0	9.9	9.5	NA
12	11.1	12.0	12.6	11.5	NA	11.5	14.5	13.3	12.5	12.6	12.6	12.0	NA
13	13.0	12.8	12.4	13.7	NA	13.0	12.2	11.5	11.6	11.4	11.0	11.4	12.2
14	10.6	10.3	10.3	10.9	10.3	11.1	11.6	11.0	NA	10.6	10.4	11.1	11.5
15	11.5	9.8	9.5	10.9	13.5	12.9	11.3	10.0	10.2	11.8	11.8	11.3	9.2
16	9.4	10.0	11.0	12.1	10.5	11.0	11.9	12.2	NA	12.5	10.9	NA	10.2
17	10.9	11.5	10.2	10.9	11.0	10.8	10.7	10.0	10.3	12.3	10.0	10.5	10.8
18	14.0	13.0	12.8	12.5	12.7	13.0	13.4	12.9	14.3	13.4	NA	13.4	13.0
19	11.8	11.9	11.4	11.8	11.6	11.9	11.5	11.8	11.8	8.5	11.0	11.1	NA
20	12.2	11.3	10.9	10.3	10.8	NA	10.8	10.9	11.2	11.0	9.9	10.6	12.3
21	12.7	12.7	11.7	11.0	12.0	12.5	11.6	11.3	10.9	11.7	NA	11.4	11.7
22	13.7	14.8	15.4	14.9	14.8	14.7	14.8	13.5	NA	12.9	12.0	NA	12.3

S3 Table. Monthly averages of hemoglobin levels [g/dl] of the study patients within the study period.

Patient	Baseline	Month 1	Month 2	Month 3	Month 4	Month 5	Month 6	Month 7	Month 8	Month 9	Month 10	Month 11	Month 12
23	14.5	14.0	13.9	NA	12.9	12.5	NA	11.5	NA	11.9	11.8	11.9	12.1
24	11.2	11.1	11.2	11.5	11.5	12.2	NA	12.8	12.8	12.7	12.4	12.3	12.4
25	9.6	10.2	10.5	10.9	11.2	10.8	11.5	11.1	11.0	11.3	11.6	11.0	11.6
26	12.1	12.7	NA	11.5	NA	12.6	12.3	NA	12.1	11.2	10.0	9.8	10.3
27	11.7	12.4	13.0	13.0	12.3	10.6	10.7	11.3	11.5	NA	11.6	11.7	11.2
28	11.8	12.3	11.6	NA	12.4	12.4	12.6	12.4	12.6	12.6	12.8	11.4	10.9
29	10.4	10.2	10.8	11.6	11.2	11.0	NA	11.0	11.4	11.3	11.4	10.9	12.0
30	11.6	11.0	11.6	11.7	11.4	11.0	11.2	11.3	11.7	11.3	NA	12.7	12.4
31	10.3	11.7	12.0	11.9	10.0	9.7	10.0	9.7	10.7	10.6	11.7	11.9	10.9
32	10.4	11.1	12.3	13.1	12.7	NA	12.2	11.5	11.8	12.0	12.2	12.4	11.7
33	10.5	11.1	12.2	13.0	11.6	10.3	NA	11.0	12.0	11.8	11.9	11.3	NA
34	10.4	10.1	10.4	10.3	10.4	9.6	9.4	9.7	10.2	10.9	11.8	11.0	NA
35	10.4	11.6	10.8	11.9	NA	11.0	12.1	11.1	10.0	10.8	11.4	NA	12.4
36	10.8	10.6	NA	11.6	NA	13.8	13.2	11.1	10.1	10.6	11.7	12.8	12.2
37	12.4	12.7	11.6	11.0	11.6	12.3	10.8	11.7	11.7	11.5	11.6	11.9	NA
38	11.9	11.0	12.3	11.3	11.1	11.2	11.8	11.8	11.6	12.5	NA	12.2	12.5
39	10.4	11.2	10.3	10.1	9.8	9.8	NA	10.9	10.1	10.2	10.8	10.8	10.1
40	9.6	9.5	9.6	9.4	9.2	10.3	NA	11.6	11.6	11.8	12.4	11.8	11.3
41	14.0	14.0	NA	13.5	NA	13.2	13.4	13.6	12.5	13.9	13.8	13.1	12.8
42	11.8	13.0	13.0	12.1	11.6	10.0	11.8	12.3	11.0	11.4	11.9	11.9	NA
43	10.4	NA	10.6	10.6	10.2	10.6	11.0	11.4	11.7	11.8	10.9	10.6	10.9
44	12.8	12.9	12.1	11.0	11.9	12.8	11.8	12.2	12.0	12.0	12.6	12.7	NA
45	10.4	10.5	11.2	10.6	NA	10.5	11.0	11.4	12.0	12.2	11.8	11.9	NA

S3 Table. Monthly averages of hemoglobin levels [g/dl] of the study patients within the study period.

Patient	Baseline	Month 1	Month 2	Month 3	Month 4	Month 5	Month 6	Month 7	Month 8	Month 9	Month 10	Month 11	Month 12
46	11.4	10.8	11.6	12.6	11.7	12.7	11.8	11.1	11.3	11.3	11.7	11.5	NA
47	13.3	13.3	13.2	13.7	13.5	NA	13.1	13.5	13.0	13.5	12.4	12.3	12.3
48	11.1	10.8	NA	11.4	10.8	10.6	10.3	NA	10.0	11.8	12.1	12.5	NA
49	13.0	13.8	13.5	12.1	NA	14.2	13.3	13.6	12.9	14.1	12.8	12.5	NA
50	11.4	12.3	13.0	12.2	12.6	12.9	NA	12.6	13.4	13.6	11.3	10.4	10.1
51	11.5	10.0	9.7	10.6	11.6	13.0	NA	12.6	9.4	8.0	NA	13.1	11.7
52	13.7	13.1	11.1	11.1	11.7	13.1	13.1	12.4	NA	10.4	11.0	11.9	12.6
53	11.3	11.7	12.9	13.0	12.8	12.0	13.0	12.8	12.9	13.9	NA	13.1	13.8
54	10.0	10.6	11.1	11.2	11.2	NA	11.8	11.5	11.2	9.7	10.2	11.4	12.0
55	13.1	12.2	12.6	14.0	13.8	13.8	14.4	13.6	NA	13.3	13.6	13.1	12.8
56	11.8	12.0	11.7	12.1	11.8	11.7	11.6	12.3	NA	11.5	11.1	11.9	10.6
57	13.2	13.1	13.6	NA	14.0	14.4	14.3	14.1	14.2	14.4	14.9	15.1	15.4
58	12.7	13.8	14.5	13.4	NA	13.6	12.9	10.8	13.2	13.4	12.1	12.6	11.8
59	10.2	10.5	11.6	12.4	12.2	11.4	11.4	11.4	10.7	NA	10.2	11.5	11.5
60	12.1	12.2	12.7	13.0	13.4	12.6	12.8	12.6	11.9	11.9	NA	12.4	13.0
61	13.6	12.5	11.6	9.6	10.1	10.6	NA	11.5	12.2	11.8	10.6	11.0	10.0
62	12.7	10.4	11.8	13.6	14.8	15.1	15.0	15.3	15.4	14.6	NA	14.8	14.4
63	11.2	11.3	11.7	12.0	11.6	NA	12.6	11.8	11.4	11.9	12.8	11.8	11.9
64	13.3	11.6	11.3	12.3	10.6	11.5	11.6	11.8	13.1	11.7	11.0	10.4	NA
65	12.2	12.0	NA	12.9	NA	12.0	11.6	12.3	12.5	12.6	12.5	11.5	12.2
66	13.2	12.1	12.7	12.8	13.4	14.4	14.6	12.1	13.4	NA	14.1	NA	13.5
67	12.0	NA	10.7	10.9	11.3	NA	11.5	11.1	10.4	11.5	11.4	11.7	10.8
68	8.1	NA	10.5	11.0	11.5	11.6	11.2	12.3	11.4	11.0	10.6	10.4	10.3

S3 Table. Monthly averages of hemoglobin levels [g/dl] of the study patients within the study period.

Patient	Baseline	Month 1	Month 2	Month 3	Month 4	Month 5	Month 6	Month 7	Month 8	Month 9	Month 10	Month 11	Month 12
69	11.6	11.3	10.9	11.0	11.2	12.1	11.2	10.7	11.2	12.1	11.8	11.7	10.5
70	11.2	11.0	11.3	10.7	10.8	11.4	NA	12.1	12.5	11.9	11.4	11.0	11.9
71	12.2	11.9	12.9	12.1	NA	10.2	10.1	10.0	10.6	11.0	11.0	11.2	NA
72	12.0	NA	11.5	NA	11.0	11.2	11.4	12.2	10.2	10.6	10.4	11.0	12.0
73	13.2	13.4	12.4	13.4	13.3	13.6	13.5	13.3	12.7	12.4	12.6	13.2	12.0
74	10.4	11.8	11.8	11.9	12.3	11.7	11.9	11.8	NA	12.0	11.9	11.7	11.7
75	12.5	12.5	11.6	12.4	12.6	11.8	11.3	11.0	NA	10.2	11.0	11.6	12.6
76	15.2	14.4	14.0	13.8	13.4	13.6	13.6	13.3	12.9	13.3	13.3	NA	12.8
77	11.3	12.1	12.3	NA	12.6	12.8	12.5	12.7	12.1	12.3	11.8	10.0	10.2
78	12.1	11.9	12.2	12.2	12.0	NA	12.6	12.9	13.8	14.0	13.6	14.7	13.6
79	14.8	15.0	14.8	15.3	16.3	13.6	13.9	15.5	14.8	15.0	13.2	NA	14.2
80	12.9	11.5	11.7	11.7	12.6	10.3	10.2	10.6	11.7	12.8	12.6	11.7	NA
81	11.4	10.6	10.5	9.6	NA	11.6	12.0	12.3	12.4	12.9	11.8	11.0	11.4
82	10.5	11.9	11.3	11.5	11.3	10.4	10.7	11.1	11.0	10.8	NA	11.5	10.9
83	12.5	12.8	12.8	12.7	11.4	NA	11.8	12.3	12.1	12.3	12.5	12.5	12.3
84	13.4	13.1	NA	12.7	12.2	13.4	13.0	13.2	13.3	12.4	9.7	NA	9.6
85	11.3	11.5	12.5	11.7	10.7	12.3	10.4	10.2	9.9	9.9	NA	10.3	11.5
86	12.2	11.4	12.4	11.1	12.7	12.3	11.8	NA	11.7	11.7	12.3	10.2	11.0
87	11.5	11.2	11.8	NA	11.6	11.1	11.5	11.9	12.4	12.2	12.3	12.7	11.5
Post-HDF													
1	12.7	11.2	11.0	11.1	11.0	12.2	11.4	9.5	10.0	11.2	10.7	10.4	9.2
2	10.9	10.6	11.1	11.5	11.5	10.6	11.6	11.9	10.1	10.4	10.2	10.3	12.3
3	13.0	12.9	13.5	NA	12.6	12.9	12.8	12.2	12.4	13.0	13.2	12.9	9.8

S3 Table. Monthly averages of hemoglobin levels [g/dl] of the study patients within the study period.

Patient	Baseline	Month 1	Month 2	Month 3	Month 4	Month 5	Month 6	Month 7	Month 8	Month 9	Month 10	Month 11	Month 12
4	13.5	10.9	11.9	11.4	11.6	12.5	11.1	12.4	NA	10.9	11.4	11.8	11.4
5	13.0	12.9	NA	12.3	9.1	10.4	11.6	12.3	11.9	11.6	11.7	11.3	11.0
6	12.8	NA	10.5	10.0	10.7	10.6	11.3	10.5	9.3	NA	11.5	11.5	12.3
7	11.2	11.7	NA	11.0	10.1	11.2	12.4	12.2	11.3	12.2	12.0	11.8	11.4
8	11.8	11.8	11.4	11.1	12.0	NA	12.7	12.6	12.9	12.2	12.0	11.9	12.7
9	13.2	13.6	13.1	13.6	14.4	14.2	14.1	13.7	14.7	14.0	14.9	14.8	14.5
10	12.0	11.6	11.4	11.5	11.6	11.2	10.5	12.0	NA	11.2	11.6	11.7	11.3
11	12.5	12.3	12.2	11.9	12.0	NA	11.6	11.0	NA	11.9	11.5	11.9	11.8
12	14.5	14.4	14.2	14.2	14.5	14.0	13.1	12.2	NA	11.7	11.4	11.4	11.7
13	11.0	10.4	11.4	11.4	NA	10.5	10.2	9.8	10.9	11.4	11.0	11.8	11.7
14	12.9	12.4	11.7	12.7	12.8	12.3	12.3	12.3	NA	11.7	11.2	11.5	9.4
15	10.8	10.2	10.2	12.8	12.2	9.4	7.7	8.5	10.9	12.3	11.1	NA	10.8
16	13.4	13.3	12.6	12.2	11.8	11.7	12.4	12.1	11.5	11.9	11.6	NA	9.4
17	11.9	NA	11.0	10.7	10.3	10.4	NA	10.1	10.3	11.2	11.7	11.9	11.4
18	10.1	11.2	11.6	10.8	11.9	11.5	NA	10.9	11.0	12.4	12.5	10.8	11.9
19	13.7	11.6	11.0	12.5	12.0	NA	13.3	14.5	12.8	14.0	12.1	NA	10.9
20	11.5	10.3	11.0	11.5	11.1	12.1	9.9	NA	10.1	10.4	10.9	11.1	11.8
21	13.1	13.5	NA	13.2	12.5	12.0	12.5	12.0	11.9	11.2	11.3	11.3	11.5
22	10.6	11.1	NA	11.1	12.0	11.4	10.9	10.5	11.6	11.2	11.4	11.3	11.6
23	11.3	11.3	NA	11.7	11.9	11.9	11.9	11.9	11.5	11.1	10.7	10.5	10.3
24	11.5	11.7	11.7	11.1	11.2	11.2	11.5	10.7	11.3	12.1	11.3	11.8	11.1
25	12.3	11.8	12.0	11.8	10.7	11.1	NA	11.9	11.5	11.3	11.3	11.9	10.4
26	11.2	12.1	10.9	11.8	11.9	11.5	11.2	11.8	12.1	NA	8.3	9.1	9.1

S3 Table. Monthly averages of hemoglobin levels [g/dl] of the study patients within the study period.

Patient	Baseline	Month 1	Month 2	Month 3	Month 4	Month 5	Month 6	Month 7	Month 8	Month 9	Month 10	Month 11	Month 12
27	12.0	11.8	12.1	11.5	11.8	12.9	NA	12.6	11.8	NA	11.0	11.1	10.8
28	9.6	10.0	9.1	9.1	10.0	11.3	12.9	9.1	9.7	9.9	10.4	10.8	11.0
29	12.0	11.5	11.7	11.8	12.5	11.8	11.6	11.8	NA	12.3	11.8	11.2	11.4
30	11.8	12.5	12.7	11.6	10.0	10.8	10.8	9.9	NA	10.0	10.1	11.1	11.4
31	11.6	12.2	10.4	10.4	10.0	11.8	12.1	NA	12.0	10.6	11.3	12.3	12.3
32	10.4	10.1	NA	12.3	11.8	11.4	11.1	10.8	10.7	11.0	11.3	10.3	9.7
33	10.0	11.2	10.5	9.2	10.5	11.4	NA	10.9	10.7	10.8	10.7	12.0	11.2
34	11.0	11.2	10.6	11.0	NA	11.2	11.8	11.3	11.0	10.4	10.3	10.4	10.6
35	11.5	12.5	11.2	10.9	12.1	11.8	12.4	12.2	NA	12.0	NA	11.5	11.4
36	10.4	10.3	10.6	NA	9.0	11.0	11.0	11.0	NA	11.6	11.4	11.4	11.3
37	11.6	11.4	11.1	11.2	11.0	10.2	11.0	NA	11.3	11.8	11.9	11.8	11.4
38	11.1	11.3	10.6	11.7	11.2	NA	11.5	10.2	NA	9.6	10.4	9.0	9.2
39	14.6	13.4	14.1	14.3	13.2	13.9	13.6	NA	12.9	13.5	13.3	12.2	12.9
40	12.8	11.8	12.2	12.9	NA	9.5	9.6	10.3	10.0	9.5	NA	NA	9.0
41	10.8	11.2	10.9	10.4	11.2	11.3	11.0	11.1	11.5	11.8	11.2	10.6	10.8
42	10.4	11.1	10.4	10.2	11.0	10.1	10.6	10.3	10.2	10.1	10.2	10.7	11.0
43	11.7	12.0	12.2	10.6	11.4	11.0	11.7	11.3	NA	11.1	10.8	10.8	11.0
44	11.1	10.6	NA	11.3	NA	10.3	10.0	11.7	11.1	10.7	11.4	9.3	9.1
45	11.4	11.6	10.4	11.7	11.6	10.8	10.8	10.9	10.0	10.4	10.4	NA	11.3
46	11.7	11.1	12.3	12.9	13.1	NA	11.9	10.8	NA	11.5	12.3	12.1	11.3
47	10.0	11.4	NA	11.9	11.6	10.7	9.8	10.5	11.8	12.0	11.3	10.0	10.6
48	13.3	12.9	12.7	12.4	12.2	NA	12.1	11.2	NA	11.8	12.2	11.7	12.4
49	10.6	11.1	11.0	11.1	11.8	10.8	10.6	10.0	NA	10.5	8.1	8.3	10.0

S3 Table. Monthly averages of hemoglobin levels [g/dl] of the study patients within the study period.

Patient	Baseline	Month 1	Month 2	Month 3	Month 4	Month 5	Month 6	Month 7	Month 8	Month 9	Month 10	Month 11	Month 12
50	11.9	11.9	11.1	11.5	11.0	10.7	10.9	11.1	10.8	11.7	10.0	11.2	NA
51	9.0	9.4	10.1	8.9	9.1	9.4	8.0	8.5	9.0	10.2	NA	11.0	8.1
52	11.9	11.4	11.5	11.6	10.6	10.1	10.2	NA	11.2	11.7	11.0	10.2	10.2
53	11.0	10.8	12.6	10.5	10.5	11.6	11.1	10.8	11.4	11.0	11.0	11.1	11.3
54	10.7	NA	10.9	9.9	10.0	10.0	10.0	11.0	11.0	11.6	12.1	11.3	11.7
55	10.7	10.5	10.0	9.9	11.5	11.7	11.5	11.4	10.6	NA	10.2	10.3	10.0
56	11.4	10.4	8.3	9.3	NA	9.2	11.3	12.2	12.5	11.5	11.4	NA	10.3
57	10.3	9.7	10.9	11.2	10.6	9.9	9.9	10.1	10.8	10.5	NA	11.1	11.0
58	12.3	11.7	11.0	10.6	12.3	12.4	12.1	11.8	NA	13.0	13.8	NA	15.2
59	13.1	11.7	NA	11.6	11.8	11.8	11.9	11.2	11.0	11.4	11.7	11.3	11.5
60	11.5	10.8	11.4	11.2	10.7	10.1	10.6	11.1	11.9	13.4	13.1	13.6	12.4
61	11.1	9.8	10.5	11.6	12.3	9.9	9.3	9.0	11.5	11.1	NA	11.4	11.5
62	11.5	11.1	10.5	11.1	11.5	11.4	11.5	11.5	NA	11.7	10.9	11.4	11.2
63	11.9	11.9	11.9	11.7	10.3	10.6	10.6	NA	11.9	NA	10.2	10.1	11.8
64	11.6	11.4	11.0	11.1	11.9	NA	11.1	11.5	11.7	10.8	11.0	11.7	11.8
65	14.3	13.9	13.5	13.3	14.0	14.4	13.4	13.6	12.9	NA	12.9	12.6	12.9
66	14.0	13.8	14.0	12.4	12.6	12.3	NA	12.4	13.0	13.0	12.5	12.1	12.3
67	10.4	10.0	9.8	9.6	10.0	10.1	9.9	6.6	NA	7.5	6.1	8.4	8.3
68	10.9	10.8	11.2	11.0	11.9	NA	11.9	13.1	13.5	13.5	12.6	12.0	10.5
69	12.5	12.8	12.1	11.8	11.8	12.6	12.6	11.9	NA	13.7	12.8	12.6	12.0
70	13.0	12.4	NA	12.4	12.7	13.5	13.0	13.6	12.4	NA	NA	10.9	11.6
71	14.3	13.7	13.7	15.1	15.3	13.7	15.7	14.9	NA	14.5	14.8	14.5	12.8
72	11.0	9.2	9.0	10.7	11.5	11.5	12.1	11.4	NA	10.5	NA	10.9	11.5

S3 Table. Monthly averages of hemoglobin levels [g/dl] of the study patients within the study period.

Patient	Baseline	Month 1	Month 2	Month 3	Month 4	Month 5	Month 6	Month 7	Month 8	Month 9	Month 10	Month 11	Month 12
73	11.7	NA	11.0	NA	9.8	9.1	10.3	10.5	11.8	11.4	10.9	11.0	8.6
74	11.6	13.1	12.9	11.5	11.5	10.4	NA	9.6	11.7	NA	11.4	11.2	10.5
75	11.5	11.4	11.0	NA	10.5	NA	11.5	11.4	11.6	12.0	11.6	11.9	11.7
76	12.3	11.9	NA	12.6	11.9	11.9	11.9	11.6	11.7	12.0	12.3	11.9	12.0
77	12.6	10.5	NA	10.6	11.3	11.4	11.3	12.3	11.5	12.6	11.9	12.1	11.9
78	11.5	10.9	10.0	10.7	11.3	11.7	11.4	10.7	10.3	NA	10.4	10.0	10.6
79	13.4	12.9	13.5	12.8	NA	12.7	12.7	12.2	12.5	11.8	12.4	NA	12.1
80	11.0	10.8	NA	10.6	10.8	11.0	11.0	10.8	11.8	12.5	11.2	10.4	10.2
81	12.1	11.2	12.4	11.9	12.9	13.0	NA	12.0	10.5	11.3	11.2	11.0	10.5
82	10.9	10.8	11.7	12.4	11.5	10.7	10.5	11.5	12.2	11.7	10.6	NA	9.6
83	12.2	12.1	12.7	12.0	11.4	11.6	11.2	11.7	12.0	12.2	10.6	NA	11.1
84	12.9	12.5	11.4	11.2	10.4	10.4	11.8	12.3	11.7	11.8	NA	11.4	11.0
85	12.9	11.4	11.9	12.1	10.1	10.8	12.4	10.5	10.7	NA	11.1	NA	12.3
86	11.5	12.1	12.3	12.5	12.7	11.9	12.0	12.5	11.8	NA	12.1	11.2	11.2
87	12.0	12.1	11.6	12.4	12.5	12.1	13.3	12.1	12.4	12.3	NA	12.6	12.3
