# Supplementary material for: Effects of high-volume online mixed-hemodiafiltration on anemia management in dialysis patients
Source: PLoS One. 2019 Feb 22;14(2):e0212795. doi: 10.1371/journal.pone.0212795 (PMC6386285; doi:10.1371/journal.pone.0212795)
Supplement: S4 Table — (PDF) [file pone.0212795.s009.pdf]

S4 Table. Total monthly ESA consumption [IU] of the study patients within the study period.

Patient	Baseline	Month 1	Month 2	Month 3	Month 4	Month 5	Month 6	Month 7	Month 8	Month 9	Month 10	Month 11	Month 12
Mixed-HDF													
1	4000	10000	8000	10000	6000	8000	4000	10000	8000	12000	12000	6000	2000
2	0	0	0	0	0	0	0	0	0	0	0	0	0
3	2000	4000	4000	2000	0	6000	0	4000	2000	4000	4000	0	0
4	6750	6750	6750	0	0	0	0	6750	6750	6750	6750	6750	6750
5	48000	32000	32000	32000	32000	16000	16000	32000	32000	32000	16000	12000	24000
6	24000	16000	16000	12000	12000	8000	8000	8000	6000	8000	8000	6000	4000
7	0	0	0	0	0	0	0	0	0	0	0	0	0
8	2000	2000	2000	2000	0	0	0	6000	0	8000	8000	4000	0
9	0	0	0	0	0	0	0	0	0	0	0	0	0
10	30000	10000	10000	30000	30000	40000	30000	30000	40000	40000	40000	20000	40000
11	12000	6750	16875	0	45000	45000	45000	33750	0	33750	0	33750	48000
12	8000	8000	4000	2000	6000	2000	2000	0	0	0	0	0	4000
13	0	0	0	0	0	0	0	0	0	0	0	0	0
14	0	0	0	0	0	0	0	0	0	0	0	0	0
15	6000	4000	16000	16000	4000	0	0	4000	0	8000	4000	0	4000
16	16000	16000	16000	6000	6000	14000	16000	8000	8000	4000	6000	6000	8000
17	4000	2000	10000	2000	4000	4000	4000	4000	4000	4000	0	4000	2000
18	0	0	0	0	0	0	0	0	0	0	0	0	0
19	40000	40000	40000	40000	40000	40000	40000	34000	28000	12000	54000	72000	72000
20	4000	0	2000	4000	8000	4000	8000	0	0	0	16000	14000	6000
21	24000	24000	6750	11250	16875	16875	11250	11250	16875	22500	22500	22500	22500
22	0	0	0	0	0	0	0	0	0	0	0	0	0

S4 Table. Total monthly ESA consumption [IU] of the study patients within the study period.

Patient	Baseline	Month 1	Month 2	Month 3	Month 4	Month 5	Month 6	Month 7	Month 8	Month 9	Month 10	Month 11	Month 12
23	0	0	0	0	0	0	0	0	0	0	0	0	0
24	32000	32000	32000	32000	32000	32000	32000	32000	32000	12000	8000	16000	16000
25	2000	8000	8000	8000	8000	8000	8000	8000	8000	8000	8000	8000	8000
26	16000	12000	0	8000	20000	18000	12000	14000	8000	8000	16000	24000	24000
27	8000	8000	6000	2000	0	0	8000	8000	8000	4000	8000	4000	4000
28	0	0	0	0	0	0	0	0	0	0	0	0	0
29	8000	12000	12000	12000	12000	12000	12000	12000	12000	12000	12000	12000	12000
30	0	0	0	0	0	0	2000	4000	2000	2000	4000	0	0
31	48000	36000	20000	8000	24000	16000	8000	24000	32000	32000	32000	24000	16000
32	2000	24000	18000	6000	4000	6000	6000	8000	4000	8000	10000	6000	8000
33	46000	40000	40000	22000	0	12000	32000	12000	20000	12000	16000	18000	18000
34	6000	8000	8000	8000	8000	8000	16000	14000	18000	16000	12000	12000	16000
35	0	4000	16000	12000	12000	16000	4000	8000	8000	4000	0	0	0
36	0	16000	22000	18000	24000	6000	0	0	18000	24000	18000	8000	8000
37	16000	8000	8000	12000	16000	12000	12000	16000	16000	16000	16000	16000	0
38	8000	16000	11250	6750	11250	16875	16875	16875	16875	16875	11250	11250	0
39	24000	24000	24000	24000	32000	32000	32000	32000	24000	24000	32000	32000	32000
40	16000	20000	20000	20000	20000	20000	20000	6000	10000	4000	0	0	0
41	0	0	0	0	0	0	0	0	0	0	0	0	0
42	16000	4000	0	0	0	4000	8000	0	0	8000	8000	8000	0
43	12000	12000	12000	12000	26000	16000	16000	8000	16000	14000	12000	12000	6000
44	0	0	0	0	0	0	0	0	0	0	0	0	0
45	48000	64000	64000	97000	0	0	81000	81000	81000	0	0	0	36000

S4 Table. Total monthly ESA consumption [IU] of the study patients within the study period.

Patient	Baseline	Month 1	Month 2	Month 3	Month 4	Month 5	Month 6	Month 7	Month 8	Month 9	Month 10	Month 11	Month 12
46	8000	8000	8000	8000	8000	4000	0	0	0	0	0	0	0
47	0	0	0	0	0	0	0	0	0	0	0	0	0
48	6000	10000	16000	6000	12000	18000	24000	24000	18000	32000	24000	12000	6000
49	0	0	0	0	0	0	0	0	0	0	0	0	0
50	24000	24000	24000	24000	24000	24000	24000	24000	18000	4000	4000	8000	8000
51	0	0	32000	32000	32000	28000	12000	0	0	0	32000	24000	0
52	33750	0	0	33750	33750	33750	0	0	0	0	33750	0	22500
53	25000	32000	22500	0	11250	11250	11250	0	11250	6750	0	0	0
54	16000	16000	16000	8000	2000	6000	4000	2000	4000	10000	4000	16000	12000
55	0	0	0	0	0	0	0	0	0	0	0	0	0
56	24000	24000	24000	24000	24000	24000	24000	24000	18000	18000	24000	12000	24000
57	0	0	0	0	0	0	0	0	0	0	0	0	0
58	14000	0	0	0	0	0	0	10000	8000	0	0	0	0
59	18000	18000	24000	18000	0	0	0	0	0	0	18000	12000	0
60	0	0	0	0	0	0	0	0	0	0	0	0	0
61	0	0	0	6000	24000	24000	22000	22000	18000	16000	20000	26000	26000
62	0	4000	4000	0	0	0	0	0	0	0	0	0	0
63	4000	0	0	0	0	0	0	2000	0	0	0	0	0
64	0	24000	24000	6000	18000	24000	24000	24000	24000	0	6000	14000	16000
65	0	0	0	0	0	0	0	0	0	0	0	0	0
66	0	0	0	0	0	0	0	0	0	0	0	0	0
67	2000	0	2000	8000	8000	8000	8000	8000	8000	8000	8000	6000	2000
68	12000	26000	22000	24000	24000	16000	12000	12000	4000	8000	10000	16000	32000

S4 Table. Total monthly ESA consumption [IU] of the study patients within the study period.

Patient	Baseline	Month 1	Month 2	Month 3	Month 4	Month 5	Month 6	Month 7	Month 8	Month 9	Month 10	Month 11	Month 12
69	12000	18000	18000	18000	18000	18000	18000	12000	24000	24000	18000	6000	12000
70	0	0	0	0	0	0	0	0	0	0	0	0	0
71	32000	24000	32000	0	0	33750	0	32000	33750	33750	33750	33750	36000
72	32000	24000	24000	16000	24000	24000	16000	8000	16000	24000	16000	8000	16000
73	4000	0	0	0	0	0	0	0	0	0	0	0	0
74	12000	16000	16000	16000	16000	16000	16000	16000	16000	10000	8000	8000	8000
75	6750	0	6750	6750	0	0	0	6750	6750	22500	0	6750	0
76	0	0	0	0	0	0	0	0	0	0	0	0	0
77	4000	4000	2000	4000	4000	2000	0	0	0	0	0	4000	4000
78	0	0	0	0	0	0	0	0	0	0	0	0	0
79	0	0	0	0	0	0	0	0	0	0	0	0	0
80	8000	8000	8000	8000	12000	8000	16000	16000	16000	8000	0	0	8000
81	12000	16000	12000	16000	32000	32000	32000	20000	8000	8000	0	4000	12000
82	12000	8000	8000	8000	8000	10000	12000	12000	12000	12000	12000	8000	8000
83	2000	2000	2000	2000	2000	4000	4000	4000	2000	4000	4000	4000	4000
84	0	0	0	0	0	0	0	0	0	0	0	0	18000
85	14000	24000	20000	16000	18000	30000	14000	24000	30000	36000	48000	20000	16000
86	20000	30000	30000	30000	32000	18000	18000	24000	24000	24000	24000	12000	36000
87	12000	14000	16000	12000	14000	18000	20000	20000	18000	16000	14000	8000	8000
Post-HDF													
1	0	8000	10000	8000	8000	4000	2000	8000	12000	6000	8000	12000	24000
2	6000	8000	14000	2000	4000	8000	16000	0	0	12000	12000	16000	4000
3	0	0	0	0	0	0	0	0	0	0	0	0	0

S4 Table. Total monthly ESA consumption [IU] of the study patients within the study period.

Patient	Baseline	Month 1	Month 2	Month 3	Month 4	Month 5	Month 6	Month 7	Month 8	Month 9	Month 10	Month 11	Month 12
4	4000	28000	28000	12000	24000	12000	8000	24000	0	24000	32000	8000	16000
5	81000	11250	0	0	97000	0	0	81000	45000	45000	0	0	0
6	0	6000	0	24000	24000	24000	36000	10000	30000	30000	40000	20000	12000
7	12000	12000	4000	8000	12000	16000	8000	0	8000	12000	0	0	0
8	8000	0	0	0	0	0	0	0	0	0	0	0	0
9	0	0	0	0	0	0	0	0	0	0	0	0	0
10	0	0	0	0	0	0	0	24000	0	0	0	0	0
11	0	0	0	0	0	0	0	0	0	0	0	0	0
12	0	0	0	0	0	0	0	0	0	0	0	0	0
13	4000	4000	12000	12000	8000	6000	4000	10000	6000	12000	8000	4000	8000
14	0	0	0	0	0	0	0	0	0	0	0	0	8000
15	0	12000	24000	18000	0	6000	0	36000	24000	24000	12000	18000	12000
16	0	0	0	0	0	0	0	0	0	0	0	0	2000
17	20000	0	20000	10000	20000	20000	10000	40000	50000	70000	40000	40000	20000
18	60000	12E4	12E4	90000	12E4	90000	90000	12E4	12E4	90000	90000	90000	90000
19	0	0	11250	0	0	0	0	0	0	0	0	0	30000
20	12000	10000	16000	16000	16000	16000	11000	18000	21000	15000	36000	36000	31000
21	0	0	0	0	0	0	0	0	0	0	0	0	0
22	16000	24000	24000	24000	8000	16000	12000	24000	18000	0	12000	6000	6000
23	16000	16000	16000	16000	16000	16000	12000	16000	12000	4000	16000	28000	24000
24	80000	50000	38000	66000	64000	64000	26000	58000	48000	36000	32000	24000	32000
25	12000	0	0	0	0	0	10000	4000	0	4000	8000	4000	0
26	2000	0	0	0	0	0	0	0	0	0	0	16000	8000

S4 Table. Total monthly ESA consumption [IU] of the study patients within the study period.

Patient	Baseline	Month 1	Month 2	Month 3	Month 4	Month 5	Month 6	Month 7	Month 8	Month 9	Month 10	Month 11	Month 12
27	0	0	0	0	0	0	0	0	0	0	4000	8000	8000
28	36000	48000	16000	12000	16000	36000	20000	40000	40000	42000	48000	48000	40000
29	4000	6000	6000	6000	6000	4000	4000	4000	4000	2000	4000	4000	4000
30	40000	30000	0	0	0	0	0	0	0	0	0	0	0
31	32000	16000	24000	16000	32000	24000	24000	24000	24000	24000	32000	24000	24000
32	16000	8000	16000	8000	0	0	4000	8000	4000	0	0	4000	16000
33	8000	12000	12000	14000	16000	8000	16000	16000	16000	24000	16000	16000	16000
34	16000	16000	16000	16000	16000	16000	12000	8000	8000	8000	16000	16000	16000
35	16000	12000	0	8000	12000	8000	12000	4000	0	0	4000	6000	6000
36	0	0	0	0	8000	12000	8000	8000	8000	8000	8000	6000	4000
37	11250	11250	11250	11250	11250	11250	11250	11250	11250	11250	11250	11250	11250
38	16000	16000	16000	12000	16000	12000	4000	4000	8000	12000	12000	12000	12000
39	0	0	0	0	0	0	0	0	0	0	0	0	0
40	0	0	0	0	0	0	64000	64000	64000	64000	64000	0	64000
41	0	4000	8000	14000	6000	12000	12000	12000	8000	8000	4000	4000	4000
42	4000	4000	2000	4000	4000	4000	8000	8000	8000	8000	8000	8000	8000
43	24000	16000	10000	12000	16000	16000	16000	16000	16000	16000	20000	24000	22000
44	28000	24000	24000	24000	24000	30000	36000	34000	32000	36000	38000	40000	40000
45	0	0	2000	2000	2000	4000	4000	2000	6000	8000	8000	8000	8000
46	2000	4000	2000	0	0	0	0	0	0	0	0	0	0
47	12000	24000	24000	6000	0	0	24000	40000	30000	0	0	0	18000
48	0	0	0	0	0	0	0	0	0	0	0	0	0
49	0	0	0	0	0	0	0	0	0	0	0	24000	32000

S4 Table. Total monthly ESA consumption [IU] of the study patients within the study period.

Patient	Baseline	Month 1	Month 2	Month 3	Month 4	Month 5	Month 6	Month 7	Month 8	Month 9	Month 10	Month 11	Month 12
50	36000	24000	36000	24000	36000	24000	36000	36000	36000	24000	36000	48000	24000
51	16000	16000	12000	8000	10000	8000	8000	8000	10000	8000	16000	8000	16000
52	30000	40000	40000	35000	25000	35000	35000	40000	40000	35000	40000	35000	45000
53	4000	4000	4000	6000	8000	8000	8000	8000	8000	8000	8000	8000	8000
54	8000	8000	4000	8000	10000	12000	14000	16000	16000	16000	12000	12000	12000
55	2000	2000	4000	8000	8000	0	0	0	0	0	0	0	4000
56	8000	4000	32000	36000	12000	20000	30000	20000	0	0	8000	8000	20000
57	10000	18000	24000	20000	6000	12000	24000	6000	24000	24000	12000	6000	0
58	0	0	4000	12000	12000	8000	8000	8000	4000	0	0	0	0
59	0	0	0	0	0	0	0	0	4000	0	0	0	0
60	12000	8000	8000	8000	8000	4000	0	0	2000	2000	0	0	0
61	22000	54000	64000	48000	56000	48000	42000	12E4	12E4	60000	64000	64000	64000
62	8000	8000	12000	12000	12000	12000	16000	12000	16000	16000	16000	16000	12000
63	32000	32000	32000	32000	32000	32000	24000	32000	32000	0	72000	88000	96000
64	60000	60000	90000	90000	90000	60000	60000	90000	90000	12E4	90000	12E4	12E4
65	0	0	0	0	0	0	0	0	0	0	0	0	0
66	6000	0	0	0	0	0	0	0	0	0	0	0	0
67	64000	48000	48000	80000	80000	60000	40000	60000	0	40000	90000	80000	80000
68	48000	24000	48000	34000	40000	30000	8000	0	0	0	0	0	0
69	0	0	0	0	0	0	0	0	0	0	0	0	0
70	6000	0	0	0	0	0	0	0	0	0	0	0	0
71	0	0	0	0	0	0	0	0	0	0	0	0	0
72	4000	10000	20000	24000	32000	24000	24000	12000	22000	18000	28000	32000	32000

S4 Table. Total monthly ESA consumption [IU] of the study patients within the study period.

Patient	Baseline	Month 1	Month 2	Month 3	Month 4	Month 5	Month 6	Month 7	Month 8	Month 9	Month 10	Month 11	Month 12
73	24000	16000	24000	16000	24000	40000	64000	64000	48000	32000	32000	16000	0
74	0	0	0	0	0	0	0	8000	12000	0	0	0	0
75	6750	0	6750	0	0	6750	6750	0	0	0	0	0	0
76	0	0	24000	0	12000	24000	24000	48000	36000	36000	24000	12000	0
77	8000	8000	24000	48000	48000	44000	36000	40000	0	32000	0	0	0
78	0	0	0	0	0	0	0	0	0	6000	14000	16000	16000
79	0	0	0	0	0	0	0	0	0	0	0	0	0
80	16000	16000	28000	24000	24000	18000	18000	24000	18000	12000	0	6000	16000
81	0	0	0	0	0	0	0	0	0	0	0	0	0
82	2000	10000	8000	6000	2000	2000	10000	16000	6000	2000	2000	2000	6000
83	16000	16000	12000	12000	16000	16000	16000	16000	16000	16000	16000	24000	18000
84	16000	16000	16000	16000	26000	40000	44000	40000	40000	40000	20000	36000	48000
85	6000	8000	8000	8000	8000	8000	16000	6000	8000	8000	6000	6000	4000
86	12000	24000	24000	24000	24000	20000	24000	24000	22000	16000	22000	18000	18000
87	0	0	2000	0	0	0	0	0	0	0	0	0	0
