# Supplementary material for: Effects of high-volume online mixed-hemodiafiltration on anemia management in dialysis patients
Source: PLoS One. 2019 Feb 22;14(2):e0212795. doi: 10.1371/journal.pone.0212795 (PMC6386285; doi:10.1371/journal.pone.0212795)
Supplement: S5 Table — (PDF) [file pone.0212795.s010.pdf]

**S5 Table.** Used ESA and iron medications in the study.

| | **Total** | **Post-HDF** | **Mixed-HDF** |
|---|---|---|---|
| **ESA [%]** | | | |
| Darbepoetin α | 68.6 | 65.0 | 72.8 |
| Epoetin α | 18.2 | 16.4 | 20.5 |
| Methoxy polyethylene glycol-epoetin beta | 2.1 | 1.2 | 3.1 |
| Epoetin β | 6.3 | 9.2 | 3.0 |
| Epoetin z | 4.8 | 8.3 | 0.7 |
| **IRON [%]** | | | |
| Iron gluconate | 92.6 | 92.8 | 92.5 |
| Iron sucrose | 7.4 | 7.2 | 7.5 |
